# OPA1 Modulates Mitochondrial Ca^2+^ Uptake Through ER-Mitochondria Coupling

**DOI:** 10.3389/fcell.2021.774108

**Published:** 2022-01-03

**Authors:** Benjamín Cartes-Saavedra, Josefa Macuada, Daniel Lagos, Duxan Arancibia, María E. Andrés, Patrick Yu-Wai-Man, György Hajnóczky, Verónica Eisner

**Affiliations:** ^1^ Departamento Biología Celular y Molecular, Facultad de Ciencias Biológicas, Pontificia Universidad Católica de Chile, Santiago, Chile; ^2^ MitoCare Center for Mitochondrial Imaging Research and Diagnostics, Department of Pathology, Anatomy and Cell Biology, Thomas Jefferson University, Philadelphia, PA, United States; ^3^ UCL Institute of Ophthalmology, University College London, London, United Kingdom; ^4^ Moorfields Eye Hospital NHS Foundation Trust, London, United Kingdom; ^5^ Cambridge Eye Unit, Addenbrooke’s Hospital, Cambridge University Hospitals, Cambridge, United Kingdom; ^6^ John van Geest Centre for Brain Repair and MRC Mitochondrial Biology Unit, Department of Clinical Neurosciences, University of Cambridge, Cambridge, United Kingdom

**Keywords:** mitochondria, OPA1, ADOA, calcium, endoplasmic reticulum

## Abstract

Autosomal Dominant Optic Atrophy (ADOA), a disease that causes blindness and other neurological disorders, is linked to *OPA1* mutations. OPA1, dependent on its GTPase and GED domains, governs inner mitochondrial membrane (IMM) fusion and cristae organization, which are central to oxidative metabolism. Mitochondrial dynamics and IMM organization have also been implicated in Ca^2+^ homeostasis and signaling but the specific involvements of OPA1 in Ca^2+^ dynamics remain to be established. Here we studied the possible outcomes of OPA1 and its ADOA-linked mutations in Ca^2+^ homeostasis using rescue and overexpression strategies in *Opa1*-deficient and *wild-type* murine embryonic fibroblasts (MEFs), respectively and in human ADOA-derived fibroblasts. MEFs lacking *Opa1* required less Ca^2+^ mobilization from the endoplasmic reticulum (ER) to induce a mitochondrial matrix [Ca^2+^] rise ([Ca^2+^]_mito_). This was associated with closer ER-mitochondria contacts and no significant changes in the mitochondrial calcium uniporter complex. Patient cells carrying *OPA1* GTPase or GED domain mutations also exhibited altered Ca^2+^ homeostasis, and the mutations associated with lower OPA1 levels displayed closer ER-mitochondria gaps. Furthermore, in *Opa1* ^*−/−*^ MEF background, we found that acute expression of *OPA1* GTPase mutants but no GED mutants, partially restored cytosolic [Ca^2+^] ([Ca^2+^]_cyto_) needed for a prompt [Ca^2+^]_mito_ rise. Finally, *OPA1* mutants’ overexpression in WT MEFs disrupted Ca^2+^ homeostasis, partially recapitulating the observations in ADOA patient cells. Thus, OPA1 modulates functional ER-mitochondria coupling likely through the OPA1 GED domain in *Opa1* ^*−/−*^ MEFs. However, the co-existence of WT and mutant forms of OPA1 in patients promotes an imbalance of Ca^2+^ homeostasis without a domain-specific effect, likely contributing to the overall ADOA progress.

## Introduction

Mitochondria are compartmentalized and dynamic organelles that undergo multiple membrane reshaping processes (Eisner et al., 2018). Mitochondria reshaping involves mitochondrial fusion, fission, and IMM folding. The GTPase protein, OPA1, is the master regulator of IMM reshaping; controlling IMM fusion, folding, cristae biogenesis, and cristae shape (Liu et al., 2009; Song et al., 2009; Varanita et al., 2015; Glytsou et al., 2016; Hu et al., 2020). Mitochondrial cristae host OXPHOS supercomplexes and support mitochondrial and cellular metabolism (Vogel et al., 2006; Cogliati et al., 2013). The multiple functions of OPA1 rely on several domains, which include the GTPase domain and the GTPase effector domain (GED) (Landes et al., 2010).

Ca^2+^ controls OXPHOS and in turn, ATP production by activating different dehydrogenases and supporting pyruvate supply inside the mitochondria (Denton, 1990; Hajnóczky et al., 1995; Jouaville et al., 1999). Ca^2+^ is principally stored at the Endoplasmic Reticulum (ER), and its release to the cytosol is mediated through the activation of the IP_3_ receptor (IP3R) or the Ryanodine receptor (Berridge, 2016). Mitochondria can physically interact with the ER through multiple protein tethers to facilitate the local Ca^2+^ transfer from the ER to mitochondria, and cytosolic Ca^2+^ (Ca^2+^_cyto_) clearance (Rizzuto et al., 1998; Csordás et al., 1999; Csordás et al., 2006). Mitochondrial Ca^2+^ (Ca^2+^_mito_) uptake is determined by: 1) the Voltage-dependent Anion Selective Channels (VDACs) and Mitochondrial Calcium Uniporter complex (mtCU), located at the outer mitochondrial membrane (OMM) and IMM, respectively; and 2) Δψ_m,_ which is the main component of the electrochemical gradient acting as the driving force (Gunter and Sheu, 2009; Rizzuto et al., 2012; Hajnóczky et al., 2014). In addition, mitochondrial morphology is a potential modulator of Ca^2+^_mito_ homeostasis that needs further investigation (Szabadkai et al., 2004; Szabadkai et al., 2006; Kowaltowski et al., 2019).

Pathogenic mutations in *OPA1* trigger Autosomal Dominant Optic Atrophy (ADOA, MIM#165500) which causes blindness due to Retinal Ganglion Cells (RGCs) death (Delettre et al., 2000; Olichon et al., 2003; Amati-Bonneau et al., 2008). OPA1 dysfunction induces IMM fusion abolition, IMM rearrangement, cristae shape disturbance and metabolic disruption (Song et al., 2009; Patten et al., 2014). The axons of mice RGCs expressing ADOA mutants, show drastic mitochondrial depletion, due to autophagosome accumulation at axonal hillocks in a AMPK-dependent manner (Zaninello et al., 2020).

The relevance of OPA1 in Ca^2+^ homeostasis has been described in the literature with many discrepancies between different groups. Knockdown of *Opa1* in RGCs caused altered Ca^2+^_cyto_ clearance upon ER Ca^2+^ (Ca^2+^_ER_) release and excitotoxicity, suggesting that OPA1 may have a role in Ca^2+^ homeostasis and disease progress (Dayanithi et al., 2010; Kushnareva et al., 2013). However, silencing of *OPA1* in HeLa and H295R cell lines induced enhanced Ca^2+^_mito_ uptake upon IP3R activation despite low Δψ_m_ and mitochondrial morphology changes (Fülöp et al., 2011). In contrast, another group showed that *OPA1* knockdown in HeLa cells displayed diminished Ca^2+^_mito_ uptake upon IP3R stimulation with serious defects on Ca^2+^_mito_ retention capacity, associated with a decrease in cristae number (Kushnareva et al., 2013). In a different study, ADOA-derived fibroblasts from a family carrying the same *OPA1* GTPase mutation showed an enhanced Ca^2+^_mito_ uptake upon IP3R stimulation but with a wide variety of responses between patients (Fülöp et al., 2015). And, expression of ADOA-causing mutants in mice RGCs and nematode motorneurons, resulted in augmented Ca^2+^_cyto_ levels (Zaninello et al., 2021). Yet, over 400 different pathogenic *OPA1* variants have been described in the Leiden Open Variation Database, LOVD (Le Roux et al., 2019) or ClinVar Database (Landrum et al., 2018) which can affect different domains such as the GTPase and GED domain among others. Finally, it is clear that OPA1 loss alters Ca^2+^ homeostasis, but the mechanisms involved still remain elusive.

Ca^2+^ uptake across the IMM is mediated by the mtCU complex. The protein core of this highly regulated complex is composed of the mitochondrial Ca^2+^ uniporter (MCU), the essential MCU regulator (EMRE), and the dominant-negative MCU subunit (MCUb) (Baughman et al., 2011; De Stefani et al., 2011; Raffaello et al., 2013; Sancak et al., 2013). The MCU activity and Ca^2+^ threshold are controlled by the mitochondrial calcium uniporter proteins (MICU1/2/3) (Perocchi et al., 2010; Mallilankaraman et al., 2012; Csordás et al., 2013; Kamer et al., 2017; Patron et al., 2019). An interaction between OPA1 and the MCU-MICU1 complex has been recently described (Herkenne et al., 2020). Also, OPA1 interacts with the mitochondrial cristae and contact site organizing system (MICOS) complex and MICU1, suggesting a role for MICU1 on cristae junction regulation (Gottschalk et al., 2019; Tomar et al., 2019). These data suggest that the role of OPA1 on Ca^2+^ homeostasis may involve mechanisms beyond its fusion activity.

High-resolution imaging experiments have shown that cristae are dynamic (Kondadi et al., 2020) and they move throughout the mitochondria in an OPA1-dependent manner (Gottschalk et al., 2018). Cristae localized close to ER-mitochondria contacts decelerate upon IP3R stimulation probably to support ER-mitochondria Ca^2+^ transfer (Gottschalk et al., 2018), reinforcing the potential role of OPA1 on Ca^2+^_mito_ signaling.

In this work, we studied the role of OPA1 and its disease causing mutations in Ca^2+^_mito_ responses upon local or bulk Ca^2+^_cyto_ signaling. We show that OPA1 is necessary to regulate ER-to-mitochondria Ca^2+^ transfer. We also demonstrate that OPA1 GTPase and GED, which are known to cause ADOA when pathogenic mutants are present, differentially alter Ca^2+^_mito_ homeostasis, with the GED domain playing a key role in ER-to-mitochondria functional coupling. In addition, the coexistence of all studied mutant and wild-type (WT) forms of OPA1 indistinctly disrupts Ca^2+^ homeostasis, potentially contributing to ADOA disease progression.

## Results

### OPA1 is Required for Cytosolic and Mitochondrial Ca^2+^ Homeostasis

**FIGURE 1:**
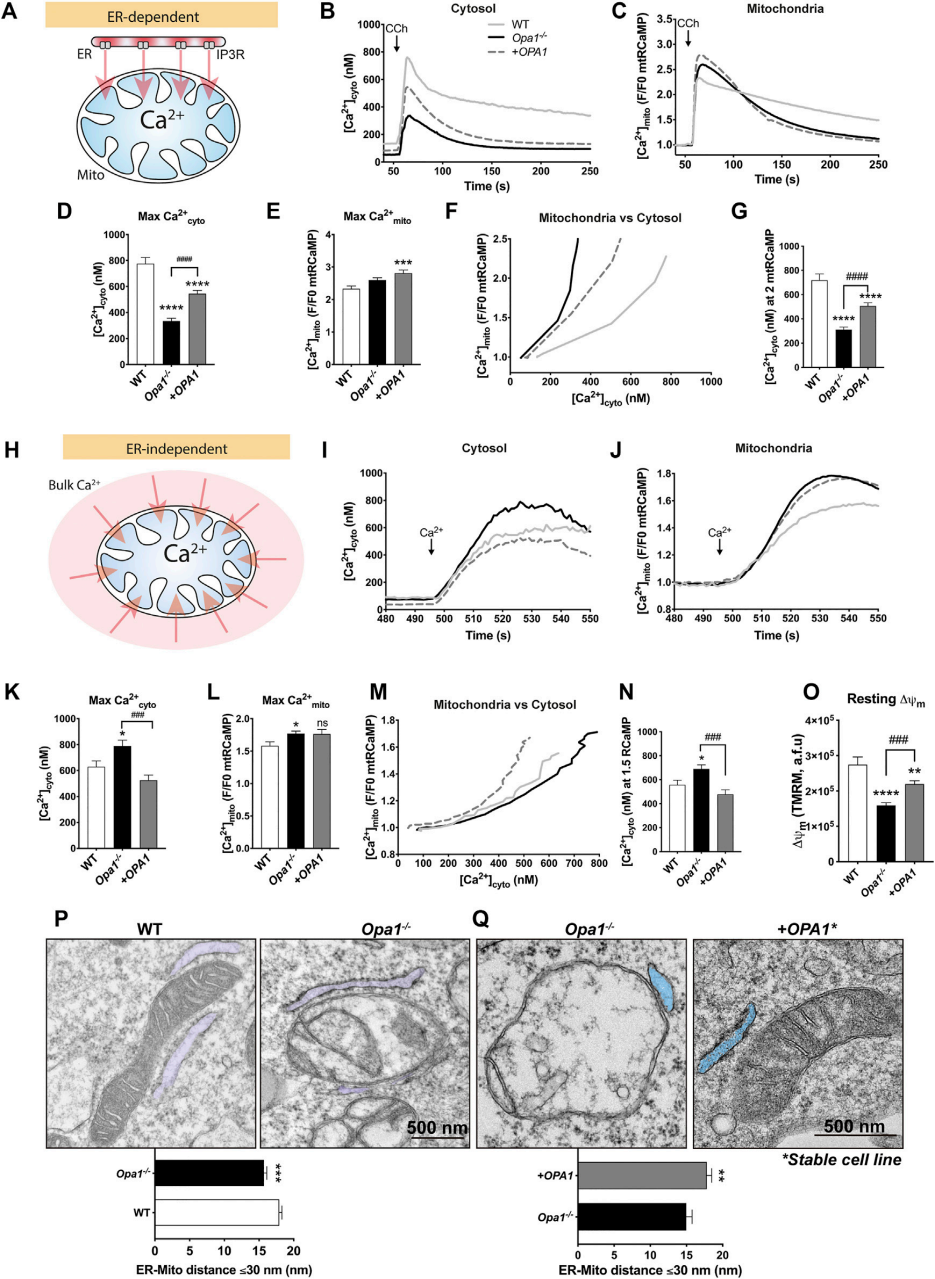
OPA1 is required for cytosolic and mitochondrial Ca^2+^ homeostasis. **(A)** ER-dependent mitochondrial Ca^2+^ uptake, schematic experimental design. WT, *Opa1* ^*−/−*^ or acute rescued *Opa1* ^*−/−*^ MEF Cells with *OPA1* WT isoform 1^*,*^ expressing mtRCaMP, and the muscarinic receptor 3 (M3R), were incubated with Fura2-AM (2 µM) for 20 min at room temperature and stimulated with 200 µM of Carbachol (CCh) to induce the release of Ca^2+^ from the ER to the cytosol via activation of IP3R **(B)** The graph shows mean traces of [Ca^2+^]_cyto_ expressed in nM (WT, *n* = 3/64; preparations/cells; *Opa1* ^*−/−*^, *n* = 3/87; *Opa1* ^*−/−*^ + *OPA1* WT, *n* = 3/100) **(C)** Mean traces of [Ca^2+^]_mito_. **(D,E)** Maximal [Ca^2+^]_cyto_ and [Ca^2+^]_mito_ amplitude upon agonist stimulation. **(F)** The graph shows mean traces of [Ca^2+^]_mito_ uptake vs IP3R-induced [Ca^2+^]_cyto_ release. **(G)** [Ca^2+^]_cyto_ at which WT, *Opa1* ^*−/−*^, and *+OPA1* WT shows 2 fold increase in [Ca^2+^]_mito_ upon CCh stimulation. **(H)** ER-independent mitochondrial Ca^2+^ uptake, schematic experimental design. Prior to stimulation with CCh, cells were washed and kept in Ca^2+^-free medium. In order to completely deplete Ca^2+^ from the ER, we first induced the stimulation of IP3R by CCh 200 µM (60s), followed by 2 µM of Thapsigargin (Tg) (200s). Finally, SOCE was triggered by 1 mM CaCl_2_ addition. **(I)** The graph shows mean traces of [Ca^2+^]_cyto_ after CaCl_2_ addition (WT, *n* = 3/78; preparations/cells; *Opa1* ^*−/−*^, *n* = 3/84; *Opa1* ^*−/−*^ *+ OPA1* WT, *n* = 3/74). **(J)** [Ca^2+^]_mito_ mean traces upon CaCl_2_ addition. **(K,L)** Maximal [Ca^2+^]_cyto_ and [Ca^2+^]_mito_ amplitude upon SOCE induction. **(M)** The graph shows mean traces of [Ca^2+^]_mito_ uptake vs SOCE-linked [Ca^2+^]_cyto_. **(N)** [Ca^2+^]_cyto_ at which WT, *Opa1* ^*−/−*^, and *+OPA1* WT shows 1.5 fold increase in [Ca^2+^]_mito_ upon SOCE induction. **(O)** Cells were incubated with TMRM 20 nM and stimulated as described above. The bar graph shows resting Δψ_m_ calculated a s the subtraction between initial fluorescence and fluorescence after FCCP addition (WT, *n* = 3/63; preparations/cells; *Opa1* ^*−/−*^, *n* = 3/105; *Opa1* ^*−/−*^ *+ OPA1* WT, *n* = 3/113). **(P)** Upper panel: TEM analysis of ER-mitochondria contacts of WT and *Opa1* ^*−/−*^ cells, representative images (ER is pseudo-colored in blue). Bottom panel: Quantification of ER-mitochondria distance ≤30 nm (WT, *n* = 3/103; preparations/mitochondria; *Opa1* ^*−/−*^, *n* = 3/99). **(Q)** Upper panel: TEM analysis of ER-mitochondria contacts of *Opa1* ^*−/−*^ and *Opa1* ^*−/−*^ + *OPA1* WT stable MEFs cells, representative images (ER is pseudo-colored in blue). Bottom panel: Quantification of ER-mitochondria distance ≤30 nm (*Opa1* ^*−/−*^, *n* = 2/90; preparations/mitochondria; *Opa1* ^*−/−*^ *+ OPA1* WT, *n* = 2/100). Data are mean ± SEM. Error bar represent SEM. (*p < 0.05vs. WT, **p < 0.01 vs. WT, ***p < 0.001 vs. WT, ****p < 0.0001 vs. WT, ^###^ *p* < 0.001 vs. *Opa1* ^*−/−*^ cells).

To test the relevance of OPA1 in Ca^2+^ homeostasis, we first used MEFs in which the mitochondrial matrix-targeted Ca^2+^-sensitive protein, mtRCaMP was expressed to monitor the [Ca^2+^] _mito_ simultaneously with [Ca^2+^] _cyto_ tracked by a Ca^2+^ sensing dye, Fura2-AM. The cells were also transfected with the muscarinic type 3 receptor (M3R) to allow Carbachol (CCh)-induced [Ca^2+^] _cyto_ transients through activation of IP3R-mediated Ca^2+^ mobilization from the ER (Figure 1A).

First, we explored the differences in the Ca^2+^ signaling between WT and *Opa1* ^*−/−*^ cells. Our data showed lower resting [Ca^2+^]_cyto_ levels (50.2 ± 2.9 nM) in *Opa1* ^*−/−*^ cells compared with WT cells (127.8 ± 7.8 nM), and upon the acute rescue with the human *OPA1* isoform 1 (*OPA1*), we found a partial restoration of the baseline (83.6 ± 5.2 nM, Supplementary Figure S1A). After CCh stimulation, we observed lower maximal [Ca^2+^]_cyto_ amplitude in *Opa1* ^*−/−*^ *cells* (333.6 ± 21.4 nM) compared with WT cells (775.9 ± 47.6 nM). Moreover, the exogenous expression of *OPA1* partially rescued the [Ca^2+^]_cyto_ signal (544.6 ± 25.8 nM, Figures 1B,D).

The resting [Ca^2+^]_mito_ was elevated in *Opa1* ^*−/−*^ cells*.* This was also detected by an alternative Ca^2+^_mito_ sensor, mtCEPIA3. In addition, *OPA1* rescue further increased resting [Ca^2+^]_mito_, suggesting that acute restoration of OPA1 to mitochondria is insufficient to lower basal [Ca^2+^]_mito_ to WT levels (Supplementary Figures S1B–E). The baseline-normalized agonist-stimulated [Ca^2+^]_mito_ transients showed no differences between WT and *Opa1* ^*−/−*^ cells, whereas the *OPA1*-rescued MEFs showed an increased [Ca^2+^]_mito_ (Figures 1C,E). Interestingly, after reaching the maximum [Ca^2+^]_mito_ amplitude, *Opa1* ^*−/−*^ cells exhibited faster decay kinetics than WT cells, however, this decay in the [Ca^2+^]_mito_ is not rescued by acute expression of *OPA1*.

To further explore the correlation between [Ca^2+^]_mito_, and IP3-linked Ca^2+^ release, the two variables were plotted against each other (Figure 1F). The graph shows that *Opa1* ^*−/−*^ cells needed less [Ca^2+^]_cyto_ to induce Ca^2+^_mito_ uptake than WT cells, which can be partially rescued by the expression of *OPA1* (Figure 1G). To test the possibility of mitochondrial Ca^2+^ uptake capacity saturation, we plotted the maximal [Ca^2+^]_mito_ vs the maximal [Ca^2+^]_cyto_ in single WT cells. As shown in Supplementary Figure S1F, there is a positive correlation between the [Ca^2+^]_cyto_ peak and the [Ca^2+^]_mito_ peak, confirming that the mitochondrial Ca^2+^ uptake or the Ca^2+^ sensor was not saturated in the range of the responses. Moreover, lower [Ca^2+^]_cyto_ was needed to induce maximal [Ca^2+^]_mito_ (Supplementary Figure 1G). In summary, the [Ca^2+^]_cyto_ records indicate lesser ER Ca^2+^ content/release, whereas the [Ca^2+^]_mito_ -[Ca^2+^]_cyto_ relationship seems to support more effective decoding of the IP3R-mediated Ca^2+^ release by mitochondrial Ca^2+^ uptake in *Opa1* deficient cells than that in the control cells.

To evaluate the ER Ca^2+^ content/release, in a Ca^2+^ free extracellular medium, we stimulated the cells with Thapsigargin (2 µM) to discharge ER Ca^2+^ by blocking the ER-Ca^2+^ re-uptake through the Sarcoendoplasmic Reticulum Calcium-ATPase (SERCA). To follow the Ca^2+^ that the mitochondria face upon Ca^2+^_ER_ release, the cells were transfected with an outer mitochondrial membrane-targeted Ca^2+^ sensor (OMMRCaMP). Both [Ca^2+^]_cyto_ and [Ca^2+^]_OMM_ responses showed a decreased Ca^2+^ release from ER (Supplementary Figures S1H–K), suggesting that the free Ca^2+^_ER_ content is attenuated in the *Opa1* ^*−/−*^ cells and this is not reverted by the acute expression of OPA1.

To further test the ER Ca^2+^ content, in a Ca^2+^ free extracellular medium, we first stimulated the cells with CCh to induce IP3R-mediated release of Ca^2+^ from the ER, and then, we added Thapsigargin to discharge the residual ER Ca^2^ (Supplementary Figure S1L). The [Ca^2+^]_cyto_ responses confirmed that ER Ca^2+^ content is reduced in the *Opa1* ^*−/−*^ cells than that in the WT. Acute expression of *OPA1* failed to rescue the ER Ca^2+^ content. (Supplementary Figures S1M,N).

During CCh stimulation, mostly local ER-to-mitochondria Ca^2+^ transfer mediates the [Ca^2+^]_mito_ rise. To test if the [Ca^2+^]_mito_ increase induced by a bulk [Ca^2+^]_cyto_ increase is also altered in the *Opa1*-deficient cells, Store Operated Calcium Entry (SOCE) was induced by adding 1 mM CaCl_2_ after the ER Ca^2+^ depletion (Figure 1H; Supplementary Figure S1L). After SOCE induction, *Opa1* ^*−/−*^ cells showed an augmented [Ca^2+^]_cyto_ transient amplitude (790.7 ± 44.2 nM) compared with WT cells (629.4 ± 44.5 nM), and this effect was completely rescued upon *OPA1* expression (525.1 ± 40.0 nM, Figures 1I,K). The corresponding [Ca^2+^]_mito_ responses were slightly but significantly augmented in *Opa1* ^*−/−*^ cells compared with WT but similar to that in the *OPA1* rescued cells (Figures 1J,L). However, plotting [Ca^2+^]_mito_ vs. [Ca^2+^]_cyto_ revealed higher [Ca^2+^]_cyto_ requirement to induce [Ca^2+^]_mito_ in *Opa1* ^*−/−*^ cells than WT or rescued cells (Figures 1M,N; Supplementary Figure S1O). Thus, *Opa1* ^*−/−*^ cells have an advantage in the mitochondrial response to IP3R-mediated Ca^2+^ release from the ER but they have a disadvantage in the response to the SOCE-mediated global [Ca^2+^]_cyto_ increase.

To test if some of the differences between WT, *Opa1* ^*−/−*^, and rescued cells might result from a difference in the driving force for the mitochondrial Ca^2+^ uptake, we studied mitochondrial membrane potential (Δψ_m_) by a potentiometric dye, TMRE. Consistent with Fülop *et al* (Fülöp et al., 2011), we found that *Opa1* ^*−/−*^ cells exhibited lower resting Δψ_m_ than WT cells, which was partially rescued by *OPA1* (Figure 1O). This might be a reason for the relatively weak [Ca^2+^]_mito_ response by the *Opa1* ^*−/−*^ cells to the enhanced SOCE.

We next tested if a difference in the mitochondrial mass or mtCU abundance/composition might explain the differential [Ca^2+^]_mito_ responses in the *Opa1* ^*−/−*^ cells and the controls. We found that both, *Opa1* ^*−/−*^ and *OPA1* rescued cells exhibit elevated levels of mitochondrial mass markers (Supplementary Figures S2A,B). We also found that *Opa1* ^*−/−*^ MEFs showed no changes in the main mtCU components after mitochondrial mass normalization (Supplementary Figures S2C,D).

Finally, we tested the ER-mitochondrial spatial relationship and mitochondrial ultrastructure as a potential source of the differences in the [Ca^2+^]_mito_ responses by transmission electron microscopy (TEM). Our results showed IMM topology perturbation and cristae compartmentalization in the *Opa1* ^*−/−*^ MEFs, as previously described (Frezza et al., 2006; Olichon et al., 2007a). Furthermore, we found significantly closer ER-mitochondria gaps in *Opa1* ^*−/−*^ cells, compared with WT cells (*Opa1* ^*−/−*^:15.71 ± 0.41 nm vs. WT: 17.92 ± 0.38 nm) (Figure 1P). To test the OPA1-specificity of our findings, we generated *Opa1* ^*−/−*^ + OPA1 WT stable MEF cells (Supplementary Figure S2E) and tested the ER-mitochondrial distance. We found increased ER-mitochondria distance in OPA1 rescued cells compared with *Opa1* ^*−/−*^ cells (*Opa1* ^*−/−*^ + OPA1: 17.81 ± 0.67 nm vs. *Opa1* ^*−/−*^: 14.98 ± 0.72) (Figure 1Q).

Thus, we conclude that the lack of Opa1 causes multiple changes in the Ca^2+^_cyto_ and Ca^2+^_mito_ homeostasis including an attenuated ER Ca^2+^ storage and enhanced SOCE. Still, the Ca^2+^_mito_ uptake response to IP3R-mediated Ca^2+^ release is enhanced, likely because of closer physical proximity between mitochondria and the ER.

### Mitochondrial Ca^2+^ Homeostasis, ΔΨm, and ER-Mitochondria Contacts Are Altered in ADOA-Derived Patient Cells Carrying *OPA1* Domain-specific Mutations

**FIGURE 2:**
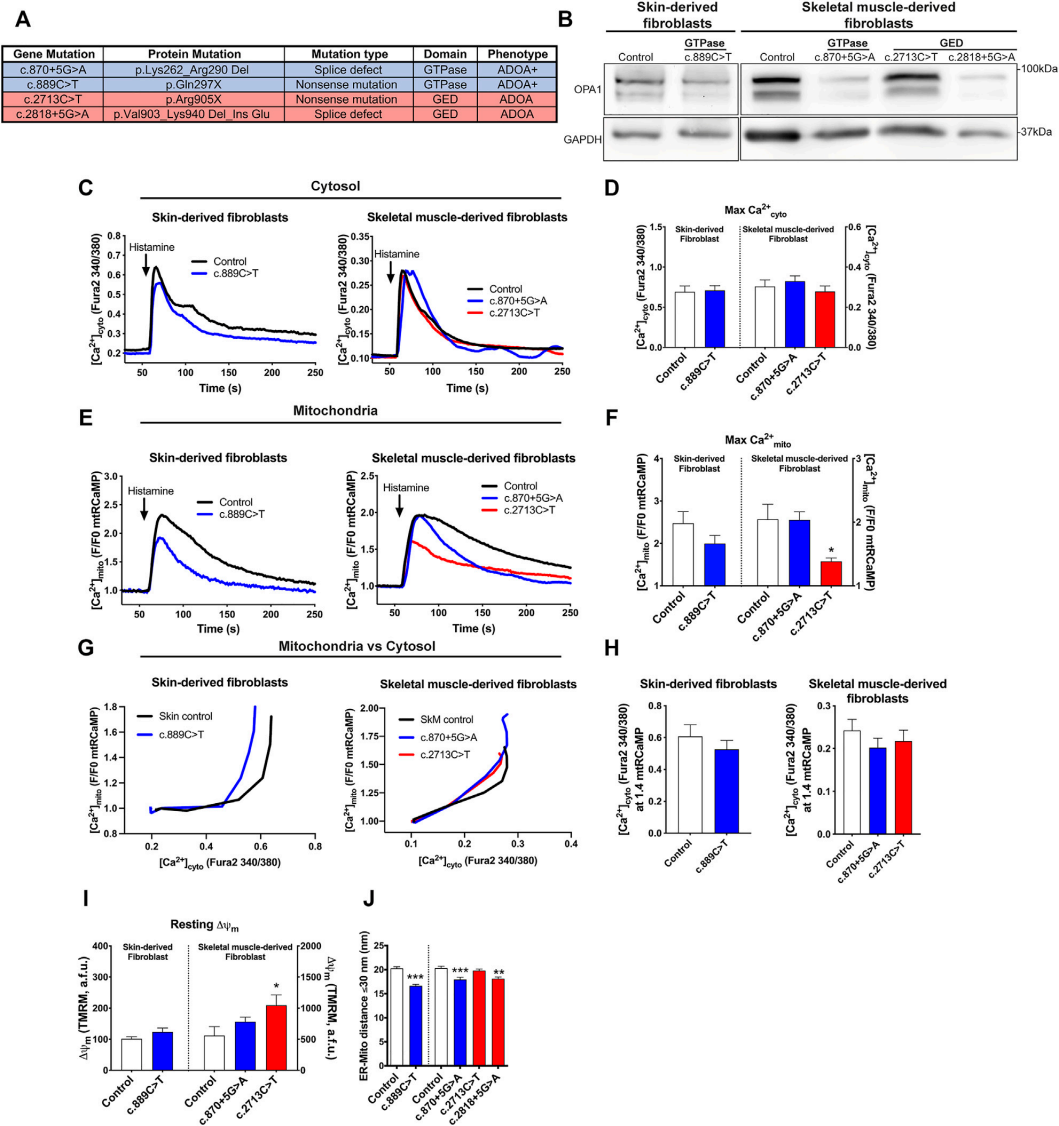
Mitochondrial Ca^2+^ homeostasis, mitochondrial membrane potential, and ER-mitochondria distance are altered in cells from ADOA patients carrying *OPA1* domain-specific mutations. **(A)** OPA1 disease-related mutations, protein product prediction, type of mutation, and clinical ADOA phenotype, evaluated in this study. **(B)** Western blot analysis of OPA1 protein abundance of the skin or skeletal muscle-derived fibroblasts from ADOA patient’s cells. **(C)** Skin or skeletal muscle ADOA-derived fibroblast expressing mtRCaMP were loaded with Fura2-AM (2 μM) for 20 min at room temperature. To induce the release of Ca^2+^ through IP3R activation, Histamine 100 μM was used. The graph show [Ca^2+^]_cyto_ expressed as Fura2-AM 340/380 mean ratio values (Skin Control, *n* = 3/12; preparations/cells; Skin *OPA1*c.889C>T, n = 3/18; SkM Control, *n* = 5/17, SkM *OPA1*c.870+5G>A, *n* = 7/21; SkM *OPA1*c.2713C>T, *n* = 4/19). **(D)** The bar chart shows maximal [Ca^2+^]_cyto_ amplitude upon agonist stimulation. **(E)** Mean traces of Skin or skeletal muscle ADOA-derived fibroblast [Ca^2+^]_mito_ upon Histamine stimulation. **(F)** The bar chart shows maximal [Ca^2+^]_mito_ uptake upon agonist stimulation expressed in F/F0 to mtRCaMP. **(G)** The graph shows mean traces of [Ca^2+^]_mito_ uptake vs IP3R-induced [Ca^2+^]_cyto_ release. **(H)** [Ca^2+^]_cyto_ at which WT, *Opa1* ^*−/−*^, and *+OPA1* WT shows 1.4 fold increase in [Ca^2+^]_mito_ upon agonist stimulation. **(I)** Skin or skeletal muscle fibroblasts from ADOA-patients were loaded with TMRM 10 nM and Fura2-AM. Incubation and stimulation were performed as described above. At the end of the experiment, FCCP 10 µM was added to dissipate me membrane potential. The bar chart shows resting Δψ_m_. (Skin Control, *n* = 3/25; preparations/cells; Skin *OPA1c.889C>T*, *n* = 3/18; SkM Control, *n* = 3/25, SkM *OPA1*c.870+5G>A, *n* = 3/25; SkM *OPA1*c.2713C>T, *n* = 3/28) **(J)** ER-mitochondria distance analysis for all mitochondria with ER contact (≤30 nm distance) (Skin control, *n* = 2/96; preparations/mitochondria), (*OPA1*c.889C>T, *n* = 2/101), (SkM control, *n* = 3/106), (*OPA1*c.870+5G>A, *n* = 3/120), (*OPA1*c.2713C>T, *n* = 3/123), (*OPA1*c.2818+5G>A, *n* = 2/92). *p*-values for Skin fibroblasts were calculated by Mann-Whitney *U*-test. *p*-values for SkM fibroblasts were calculated using Kruskal-Wallis test. Data are mean ± SEM. Error bar represent SEM. *p < 0.05, **p < 0.01, ***p < 0.001 vs respective control condition The blue color is indicative of GTPase mutants and the red color for GED mutants.

We wanted to address if ADOA patient-derived fibroblasts carrying domain-specific heterozygous *OPA1* mutations replicate the defects in Ca^2+^ homeostasis and ER-mitochondria distance *Opa1* ^*−/−*^ cells exhibited. Fibroblasts carrying specific mutations at the GTPase domain included *OPA1* c.870+5G>A, with a deletion of the Exon8, and c.889C>T with an early stop codon at GTPase domain. Both patients presented a severe form of the disease, involving other symptoms besides optic nerve atrophy, known as ADOA+ (MIM# 125250). Also, we studied patients carrying specific mutations in GED: *OPA1* c.2713C>T leading to a stop codon and loss of the GED domain, and c.2818+5G>A with a deletion of GED coding exon 27. These patients presented symptoms restricted to the eye or plain ADOA (MIM#165500) (Figure 2A). Western blot analysis revealed that patients’ cells carrying c.889C>T, c.870+5G>A, and c.2818+5G>A mutations, exhibited lower OPA1 protein levels compared with Control cells or the c.2713C>T mutant (Figure 2B; Supplementary Figure S3A).

To evaluate intracellular Ca^2+^ signaling, we first measured resting [Ca^2+^]. Our results showed that patients’ cells have unaltered resting [Ca^2+^]_cyto_ (Figures 2C,D; Supplementary Figure S3B). Analysis of Ca^2+^_mito_ showed no differences in [Ca^2+^]_mito_ resting in patients’ cells carrying GTPase mutants. However, the GED mutant c.2713C>T showed elevated resting [Ca^2+^]_mito_ compared with control patient cells (Supplementary Figure S3C).

Then, we used histamine as an agonist to induce Ca^2+^ release from the ER through activation of the IP3R. Data in Figures 2C,D show that the patients’ cells displayed comparable maximal [Ca^2+^]_cyto_ transient amplitude than control individuals. Cells carrying GTPase mutations showed no significant changes in [Ca^2+^]_mito_ rise; however, cells carrying the GED mutant c.2713C>T displayed lower [Ca^2+^]_mito_ increase compared with control cells (Figures 2E,F). [Ca^2+^]_mito_ vs.[Ca^2+^]_cyto_ chart showed that all the ADOA patient’s cells had a leftward shift tendency in this relationship compared to control cells (Figures 2G,H; Supplementary Figure S3D).

Next, we evaluated the resting Δψ_m_ in ADOA-derived cells. Fibroblast carrying GTPase mutants showed no significant changes, but GED mutant c.2713C>T exhibited a higher resting Δψ_m_ compared with control cells, which may be a factor in the elevated resting [Ca^2+^]_mito_ levels and therefore, reduced [Ca^2+^]_mito_ transients amplitude (Figure 2I).

Finally, we tested the distance between the ER and the mitochondria. EM analysis of ADOA-derived fibroblasts revealed that cells carrying the GTPase mutants c.889C>T, c.870+5G>A and the GED mutant c.2818+5G>A exhibited significantly closer ER-mitochondria apposition, compared with control cells (Skin Control: 20.24 ± 0.35 nm, *OPA1* c.889C>T 16.6 ± 0.35 nm, SkM Control: 20.3 ± 0.39 nm, *OPA1* c.870+5G>A 17.93 ± 0.49 nm, *OPA1* c.2818+5G>A 18.09 ± 0.38 nm). Yet, we found no differences between control and GED mutant c.2713C>T carrying cells (Figure 2J; Supplementary Figure S3E). Strikingly, all the samples that exhibited closer ER-mitochondria contacts also displayed lower OPA1 protein levels (Figure 2B).

In summary, ADOA-derived fibroblasts showed no defects on [Ca^2+^]_cyto_ transients unlike *Opa1* ^*−/−*^ cells. However, we found that all the studied mutants needed lesser Ca^2+^_cyto_ to trigger a Ca^2+^_mito_ rise, consistent with the observations in *Opa1* ^*−/−*^ cells. Interestingly, the patients’ cells showed a correlation between OPA1 protein levels and ER-mitochondria distance. Thus, despite the distinct severities of ADOA disease, caused by GTPase and GED mutants, both kinds of aberrant proteins lead to similar Ca^2+^ homeostasis dysregulation.

### OPA1 GED Domain Determines Functional ER-to-Mitochondrial Coupling

Given that patients’ cells might carry genetic or environmental adaptations, we next studied the domain-specific effects of *OPA1* mutants in an *Opa1* ^*−/−*^ background. For this, we generated ADOA-causing *OPA1* GTPase and GED domain-specific mutants, matching the patients’ genotype (Supplementary Figure S4A). We observed that different mutants show distinct protein levels, consistent with our observation in the patients’ cells (Figure 2A). Particularly, mutants carrying splicing defects, such as *OPA1* c.870+5G>A and c.2818+5G>A, display lower protein levels than WT OPA1, suggesting defects in protein stability.

**FIGURE 3:**
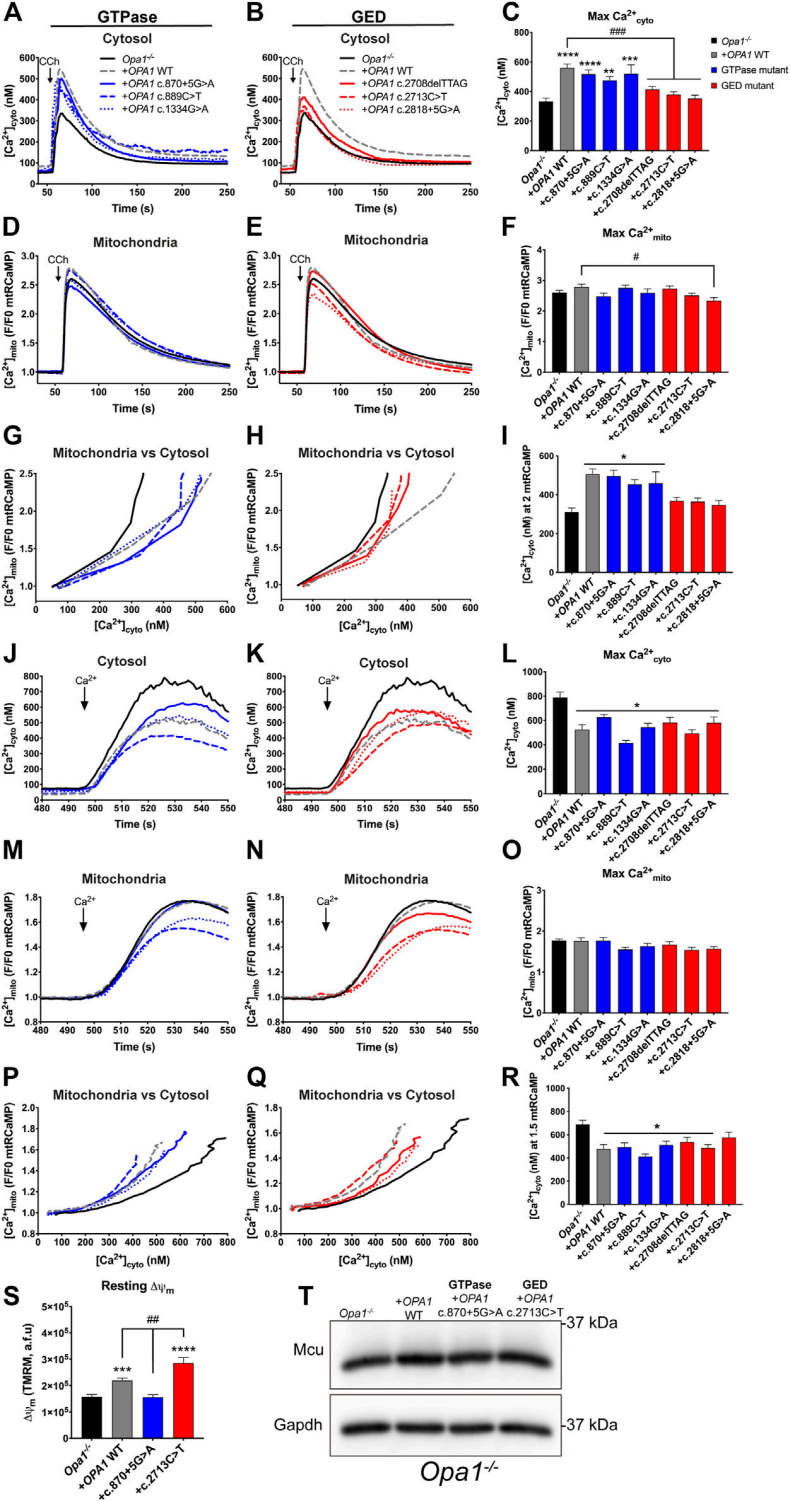
OPA1 GED domain is required effective ER-to-mitochondrial Ca^2+^ transfer. **(A,B)** ER-dependent Ca^2+^ release. *Opa1^−/−^* MEF cells expressing *OPA1* mutants, mtRCaMP, and the muscarinic receptor 3 (M3R), were incubated with Fura2-AM as described, and stimulated with 200 µM of Carbachol (CCh) to induce the release of Ca^2+^ from the ER to the cytosol via activation of IP3R. Graph show mean traces of [Ca^2+^]_cyto_ expressed in nM. **(A)** Shows mean traces of GTPase mutants and **(B)** shows mean traces of GED mutants of [Ca^2+^]_cyto_ transients, respectively (*Opa1^−/−^* MEF, *n* = 3/87; *Opa1^−/−^* *+ OPA1* WT, *n* = 3/100; *Opa1^−/−^* *+ OPA1*c.870+5G>A, *n* = 3/100; *Opa1^−/−^* *+ OPA1*c.889C>T, *n* = 3/55; *Opa1^−/−^* *+ OPA1*c.1334G>A, *n* = 3/35; *Opa1^−/−^* *+ OPA1*c.2708delTTAG, *n* = 3/69; *Opa1^−/−^* + *OPA1c*.2713C>T, *n* = 3/109; *Opa1^−/−^* + *OPA1*c.2818+5G>A, *n* = 3/55). **(C)** The bar chart shows maximal [Ca^2+^]_cyto_ amplitude upon agonist stimulation. **(D,E)** The graph shows mean traces of [Ca^2+^]_mito_ uptake for *OPA1* GTPase and GED mutants. **(F)** Maximal [Ca^2+^]_mito_ uptake induced upon agonist stimulation. **(G,H)** *OPA1* GTPase and GED mutants [Ca^2+^]_mito_ uptake vs IP3R-induced [Ca^2+^]_cyto_ upon agonist stimulation. **(I)** [Ca^2+^]_cyto_ at which *OPA1* GTPase and GED mutants shows 2 fold increase in [Ca^2+^]_mito_ upon agonist stimulation. **(J,K)** ER-independent Ca^2+^ transients; SOCE. *Opa1^−/−^* MEF cells expressing hOPA1 mutants, mtRCaMP, and the muscarinic receptor 3 (M3R), were incubated with Fura2-AM as described above. Prior stimulation with CCh, cells were washed and kept in a Ca^2+^ free medium. To induce the stimulation of IP3R, cells were treated with CCh 200 µM, followed with 2 µM of Thapsigargin (Tg) to completely deplete the Ca^2+^ from the ER. Then, 1 mM of CaCl_2_ was added to induce the SOCE. Graph show mean traces of [Ca^2+^]_cyto_ expressed in nM upon SOCE induction for GTPase and GED mutants (*Opa1^−/−^* MEF, *n* = 3/84; *Opa1^−/−^* *+ OPA1* WT, *n* = 3/74; *Opa1^−/−^* *+ OPA1*c.870+5G>A, *n* = 3/50; *Opa1^−/−^* *+ OPA1*c.889C>T, *n* = 3/35; *Opa1^−/−^* *+ OPA1*c.1334G>A, *n* = 3/47; *Opa1^−/−^* *+ OPA1*c.2708delTTAG, *n* = 3/38; *Opa1^−/−^* *+ OPA1*c.2713C>T, *n* = 3/54; *Opa1^−/−^* *+ OPA1*c.2818+5G>A, n = 3/36). **(L)** Maximal [Ca^2+^]_cyto_ amplitude upon SOCE induction. **(M,N)** Mean traces of [Ca^2+^]_mito_ uptake upon SOCE induction. **(O)** Maximal [Ca^2+^]_mito_ uptake upon SOCE induction. **(P,Q)** The graphs show [Ca^2+^]_mito_ uptake vs SOCE-linked [Ca^2+^]_cyto_. **(R)** [Ca^2+^]_cyto_ at which *OPA1* GTPase and GED mutants shows 1.5 fold increase in [Ca^2+^]_mito_ upon SOCE induction. **(S)** Same cells described in A were incubated with TMRM 20 nM and stimulated as described above. The bar graph shows resting Δψ_m_ calculated as the subtraction between initial fluorescence and fluorescence after FCCP addition (*Opa1^−/−^* MEF, *n* = 3/105; *Opa1^−/−^* *+ OPA1* WT, *n* = 3/113; *Opa1^−/−^* *+ OPA1*c.870+5G>A, *n* = 3/103; *Opa1^−/−^* *+ OPA1*c.2713C>T, *n* = 3/73). **(T)** Western blot analysis of Mcu protein abundance upon acute expression of *OPA1* GTPase or GED mutants in *Opa1^−/−^* MEF cells. Data are mean ± SEM. Error bars represent SEM. *p < 0.05, **p < 0.01, ***p < 0.001, ****p < 0.0001 vs. *Opa1^−/−^* condition. ^#^ *p* < 0.05, ^##^ *p* < 0.01, ^###^ *p* < 0.001, ^####^ *p* < 0.0001 vs. *Opa1^−/−^ + OPA1* WT condition. The blue color is indicative of GTPase mutants and the red color for GED mutants.

Our data showed that the acute expression of WT *OPA1* rescues decreased resting [Ca^2+^]_cyto_ levels shown by *Opa1* ^*−/−*^ cells; nevertheless, the expression of each ADOA-causing OPA1 mutants displayed resting [Ca^2+^]_cyto_ levels comparable to *Opa1* ^*−/−*^ cells. These data suggest that OPA1 integrity is critical to support a mitochondrial role in the Ca^2+^_cyto_ balance under non-stimulated conditions (Figures 3A,B; Supplementary Figure S4B). The analysis of Ca^2+^_mito_ showed that all studied *OPA1* GED mutants exhibited increased resting [Ca^2+^]_mito_ (Supplementary Figures S4C–E). Consistently, we observed the same effect in the *Opa1* ^*−/−*^ cells and the *OPA1* GED mutation c.2713C>T patient cells (Supplementary Figures S1A, S3C, respectively), suggesting that the GED region, and not the GTPase domain, may play a specific role in the regulation of [Ca^2+^]_mito_ resting levels.

Upon CCh stimulation, both WT and *OPA1* GTPase mutants rescued the maximal [Ca^2+^]_cyto_ rise; whereas, GED domain mutants showed no rescue (Figures 3A–C). In terms of maximal [Ca^2+^]_mito_ transient amplitude triggered by CCh, neither the acute expression of WT *OPA1* nor the *OPA1* mutants, showed a significant difference compared to *Opa1* ^*−/−*^ cells (Figures 3D–F). This might be a consequence of a chronic absence of Opa1 or alternatively, more than one OPA1 isoform is needed to restore Ca^2+^_mito_ homeostasis, as it has been demonstrated for mitochondrial network morphology (Del Dotto et al., 2017).

We next studied the correlation between ER-associated [Ca^2+^]_mito_ and [Ca^2+^]_cyto_ responses. Our data showed that as in *Opa1* ^*−/−*^ background, the expression of *OPA1* GED mutants, displayed a leftward shift in [Ca^2+^]_cyto_ needed to induce a [Ca^2+^]_mito_ rise compared with WT or GTPase rescued conditions (Figures 3G–I). Moreover, the levels of [Ca^2+^]_cyto_ needed to generate the maximal [Ca^2+^]_mito_ were rescued by the *OPA1* GTPase mutants, while *OPA1* mutants lacking GED displayed only a mild rescue (Supplementary Figure S4F). Thus, our data suggest that Ca^2+^ transfer to the mitochondria from local Ca^2+^_cyto_ is enhanced when the GED region or the entire protein is missing. However, this asseveration doesn’t totally match with the GTPase mutant c.889C>T, which expresses a truncated protein form due to a stop codon (Figure 2A) and showed a behavior comparable to the GTPase mutants rather than the GED mutants.

To test the OPA1 domain-specific effects on the Ca^2+^_mito_ rise prompted by bulk Ca^2+^_cyto_ increases, we turned to SOCE. Upon SOCE induction, the augmented [Ca^2+^]_cyto_ transients of the *Opa1* ^*−/−*^ cells were restored by WT *OPA1*. All the mutants also displayed [Ca^2+^]_cyto_ transients comparable with WT *OPA1* (Figures 3J–L). However, no significant effect was observed on [Ca^2+^]_mito_ upon SOCE induction in *Opa1* ^*−/−*^ or rescued cells (Figures 3M–O).

A two-variable chart was plotted to explore the role of bulk Ca^2+^_cyto_ entry on ER-independent Ca^2+^_mito_ uptake. The graphs show that upon [Ca^2+^]_cyto_ increase, *Opa1* ^*−/−*^ cells exhibited a rightward shift in [Ca^2+^]_cyto_ needed to induce [Ca^2+^]_mito_ rise, compared with the GTPase or GED mutants (Figures 3P–R). Moreover, *Opa1* ^*−/−*^ mitochondria required 790.7 ± 44.2 nM of [Ca^2+^]_cyto_ to reach the maximal [Ca^2+^]_mito_, and this was completely restored with the inclusion of either *OPA1* or the mutants, requiring lower [Ca^2+^]_cyto_ to reach the maximal [Ca^2+^]_mito_ (Supplementary Figure S4G).

Next, we measured resting Δψ_m_ to test if the driving force of Ca^2+^ uptake is altered upon the expression of some of the *OPA1* mutants. No change in resting Δψ_m_ was detected in the GTPase mutant c.870+5G>A (Figure 3S). We found the GED mutant c.2713C>T exhibited an augmented resting Δψ_m_ compared with *OPA1* WT rescue (Figure 3S), as we observed in the patient cells harboring the same mutant (Figure 2I). Thus, although WT and GTPase OPA1 mutants can restore ER-to-mitochondrial Ca^2+^ transfer, this seems independent of the cation driving force evoked by Δψ_m_. Finally, Mcu protein levels were unaltered by the acute expression of neither *OPA1* GTPase nor GED mutants (Figure 3T).

Thus, OPA1 GED region plays a critical role in ER-mitochondrial Ca^2+^ transfer, and this effect is independent of resting Δψ_m_, OPA1, or Mcu protein levels. Moreover, Ca^2+^_mito_ uptake induced by bulk Ca^2+^_cyto_ was restored by OPA1 or the mutants suggesting that the sole physical presence of the protein is sufficient to support this process, in a domain-specific independent manner.

### Overexpression of *OPA1* Mutants impairs Ca^2+^ Homeostasis

Considering the heterozygosity of the ADOA patients carrying *OPA1* mutations, we tested if the introduction of *OPA1* mutants, in the presence of native Opa1 in the WT MEF background, disrupts normal Ca^2+^ homeostasis like what was observed in the patient cells.

Our results showed that acute expression of *OPA1* mutants did not alter resting [Ca^2+^]_cyto_ (Supplementary Figure S4H). Interestingly, the GTPase mutant c.1334G>A exhibited a high resting [Ca^2+^]_mito_ when compared with WT *OPA1* overexpression (Supplementary Figures S4I–K).

**FIGURE 4:**
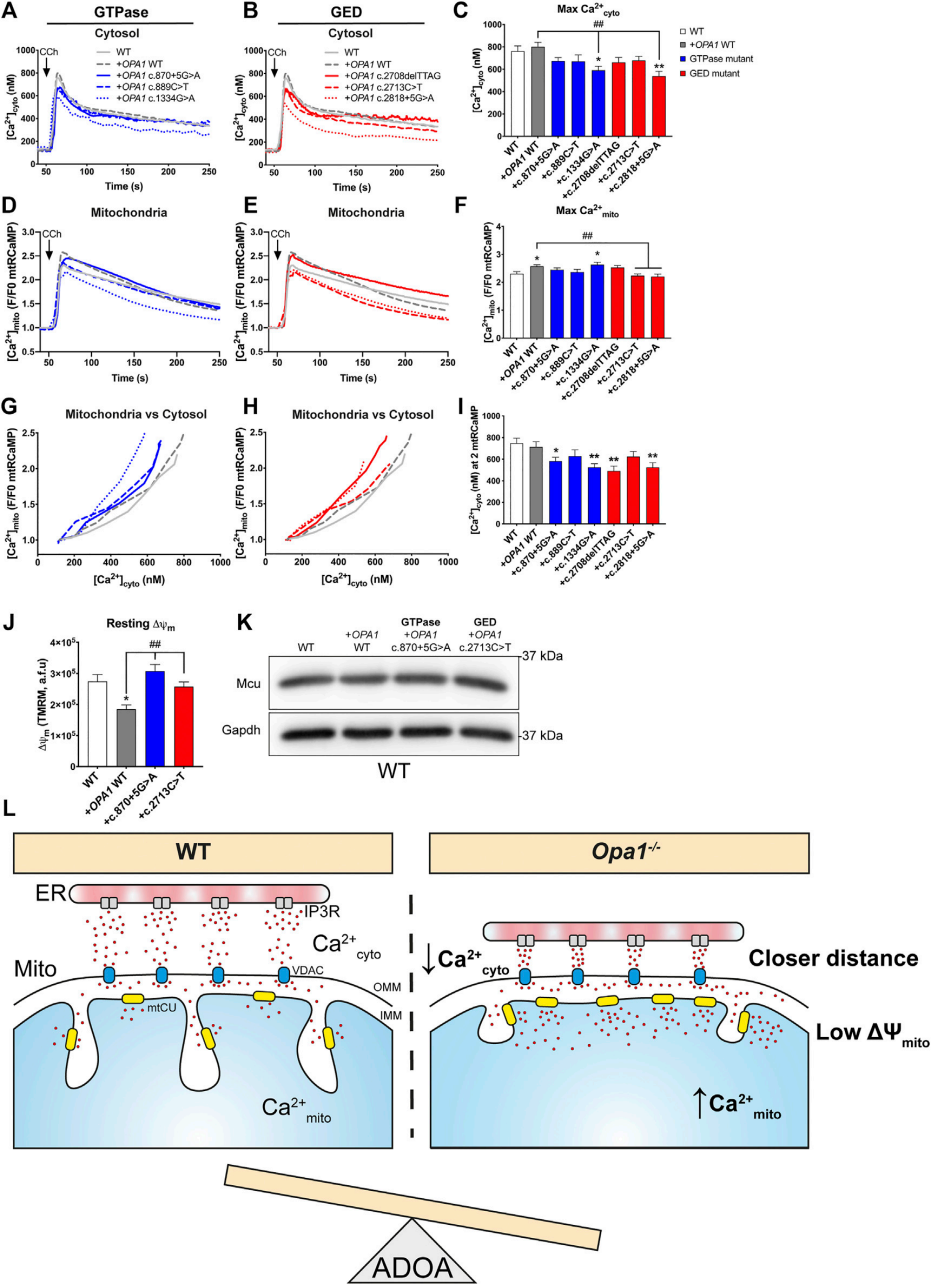
Overexpression of ADOA-causing *OPA1* mutants impairs ER-to mitochondrial Ca^2+^ transfer. **(A,B)** WT MEF cells expressing *OPA1* mutants, mtRCaMP, and the muscarinic receptor 3 (M3R), were incubated with Fura2-AM and stimulated with 200 µM of Carbachol (CCh) to induce the release of Ca^2+^ from the ER to the cytosol via activation of IP3R. Graph show mean traces of [Ca^2+^]_cyto_ expressed in nM, in WT MEF expressing GTPase and (B) in GED mutants (WT MEF, *n* = 3/64; WT + *OPA1* WT, *n* = 3/58; WT *+ OPA1*c.870+5G>A, *n* = 3/49; WT *+ OPA1*c.889C>T, *n* = 3/48; WT *+ OPA1*c.1334G>A, *n* = 3/51; WT *+ OPA1*c.2708delTTAG, *n* = 3/47; WT *+ OPA1*c.2713C>T, *n* = 3/49; WT *+ OPA1*c.2818+5G>A, *n* = 3/42) **(C)** Bar chart shows maximal [Ca^2+^]_cyto_ amplitude upon agonist stimulation. **(D,E)** The graph shows mean traces of [Ca^2+^]_mito_ uptake in *OPA1* GTPase and GED mutants, expressing WT MEF cells. **(F)** Maximal [Ca^2+^]_mito_ uptake induced upon agonist stimulation. **(G,H)** *OPA1* GTPase and GED mutants [Ca^2+^]_mito_ uptake vs IP3R-induced [Ca^2+^]_cyto_ upon agonist stimulation. **(I)** [Ca^2+^]_cyto_ at which *OPA1* GTPase and GED mutants shows 2 fold increase in [Ca^2+^]_mito_. **(J)** The same cells described in **A** were incubated with TMRM 20 nM and stimulated as described by Carbachol. The bar graph shows resting Δψ_m_ calculated as the subtraction between initial fluorescence and fluorescence after FCCP addition (WT MEF, n = 3/63; WT + *OPA1* WT, *n* = 3/117; WT *+ OPA1c.870+5G>A*, *n* = 3/159; WT *+ OPA1c.2713C>T*, *n* = 3/117; WT). **(K)** Western blot analysis of Mcu protein abundance upon acute expression of *OPA1* GTPase or GED mutants in WT MEF cells. Data are mean ± SEM. Error bar represent SEM. *p < 0.05, **p < 0.01 vs. WT condition. ^#^ *p* < 0.05, ^##^ *p* < 0.01 vs. WT + *OPA1* WT condition. The blue color is indicative of GTPase mutants and the red color for GED mutants. **(L)** Working model. Cells lacking *OPA1* exhibited closer ER-mitochondria contacts and a leftward shift in Ca^2+^_cyto_ dependence compared to WT cells evoking a more efficient ER-to-mitochondrial Ca^2+^ transfer. *OPA1* ADOA-causing mutants disrupt Ca^2+^ homeostasis inducing an *OPA1* dominant-negative phenotype, probably contributing to disease progression.

Upon CCh stimulation, we found a decreased maximal [Ca^2+^]_cyto_ amplitude in the GTPase mutant c.1334G>A when is compared with WT *OPA1* overexpression (Figures 4A–C). The same mutant exhibited an elevated maximal [Ca^2+^]_mito_ transient amplitude upon agonist stimulation (Figures 4D–F). In addition, GED mutant c.2818+5G>A exhibited a reduced maximal [Ca^2+^]_cyto_ amplitude after agonist stimulation compared to WT *OPA1* overexpression (Figure 4C). Moreover, both GED mutants c.2713C>T and c.2818+5G>A displayed a reduced [Ca^2+^]_mito_ (Figure 4F). The same reduction in [Ca^2+^]_mito_ was observed in the ADOA patient’s cells carrying the GED mutant c.2713C>T.

Correlative study of [Ca^2+^]_mito_ and [Ca^2+^]_cyto_ transients showed that overexpression of *OPA1* WT displayed no alteration in the [Ca^2+^]_cyto_ needed to trigger a [Ca^2+^]_mito_ rise. In contrast, the acute expression of most GTPase or GED mutants, prompted a leftward shift in [Ca^2+^]_cyto_ needed to induce [Ca^2+^]_mito_ rise upon agonist stimulation (Figures 4G–I). Consistently, as oppose to WT *OPA1* overexpression, the inclusion of GTPase or GED mutants lowered the maximal [Ca^2+^]_cyto_ needed to evoke maximal [Ca^2+^]_mito_ (Supplementary Figure S4L).

Evaluation of Δψ_m_ revealed that acute overexpression of *OPA1* WT decreased resting Δψ_m_ (Figure 4J). Yet, neither GTPase mutant c.870+5G>A nor GED mutant c.2713C>T overexpression altered resting Δψ_m_ in a WT MEF background. (Figure 4J). Finally, we found no differences in Mcu protein levels caused by WT or mutant OPA1 overexpression (Figure 4K).

Thus, acute expression of ADOA-causing *OPA1* mutants in the presence of endogenous Opa1 causes a dominant-negative phenotype in ER-to-mitochondria Ca^2+^ transfer.

## Discussion

We showed here that cells with low levels of Opa1 exhibit closer ER-mitochondria apposition, likely resulting in a more efficient ER-to-mitochondria Ca^2+^ transfer. This occurs without changes in the mtCU components or mitochondrial mass, and despite the reduced resting Δψ_m_ presented by the Opa1-deficient cells. In an *Opa1*-null background, ADOA-causing mutants located in OPA1 GED region perturbed ER-dependent Ca^2+^_cyto_ transients and Ca^2+^_mito_ uptake. In WT background, OPA1 mutants evoke a dominant-negative phenotype independent of the domain affected by the specific mutation possibly, contributing to ADOA disease progression (Figure 4L; Supplementary Table S1).

In different cell types, the absence of OPA1 has shown diverse results. In cultured RGCs, the principal cells affected by *OPA1* mutants causing DOA (Votruba et al., 1998), silencing of *Opa1* augmented Ca^2+^_cyto_ transients (Dayanithi et al., 2010; Kushnareva et al., 2013). Interestingly, RGCs expressing OPA1 ADOA-causing mutants, show elevated Ca^2+^_cyto_ (Zaninello et al., 2021). However, proopiomelanocortin neurons devoid of Opa1 show unaltered Ca^2+^_cyto_ transients, and reduced Ca^2+^_mito_ responses (Gómez-Valadés et al., 2021). Finally, adult cardiomyocytes of *Opa1* ^*+/−*^ mice displayed lower [Ca^2+^]_cyto_ amplitude (Chen et al., 2012; Le Page et al., 2016) or enhanced Ca^2+^_mito_ uptake (Piquereau et al., 2012). These studies suggest that OPA1 affects intracellular Ca^2+^ homeostasis, possibly, in a cell-type specific manner.

Our data in *Opa1* ^*−/−*^ cells exhibited alterations in cytosolic, ER and mitochondrial Ca^2+^ homeostasis. Resting [Ca^2+^]_cyto_ was reduced in *Opa1* ^*−/−*^, which could be explained by a decreased influx or increased efflux of Ca^2+^. The former is unlikely because a higher influx was detected upon SOCE, whereas, the latter is not consistent with our Δψ_m_ data, which suggests lower ATP levels in these cells. Also, CCh-induced [Ca^2+^]_cyto_ transient amplitude was decreased in *Opa1* ^*−/−*^ cells, caused by lower ER content, suggesting that chronic absence of Opa1 leads to an adaptation in ER Ca2+ homeostasis, that could involve IP3R or SERCA levels or activity alterations. Moreover, resting and maximal [Ca^2+^]_mito_ was augmented despite low resting Δψ_m_ during local Ca^2+^ transfer. This observation is consistent with previous reports in OPA1 silenced intact HeLa and H295R cells using a genetically encoded Ca^2+^_mito_ sensor (Fülöp et al., 2011). Conversely, another group reported *OPA1* silencing decreases maximal [Ca^2+^]_mito_ amplitude upon Histamine stimulation, using Rhod-2 as a Ca^2+^_mito_ sensor in intact cells (Kushnareva et al., 2013). These opposite results can be attributed to the different technical approaches. The mitochondria-specific nature of [Ca^2+^]_mito_ transients studied by genetic encoded Ca^2+^-sensitive proteins, provides strong pieces of evidence to confirm that both acute (Fülöp et al., 2011), and chronic absence of OPA1 studied in this work lead to augmented Ca^2+^_mito_ uptake. Hence, our study strengths the idea of OPA1 as a key molecular modulator in ER-to-mitochondria Ca^2+^ transfer.

The reduced [Ca^2+^]_cyto_ needed to induce a [Ca^2+^]_mito_ rise shown by *Opa1* ^*−/−*^ cells likely reflects enhanced ER-to-mitochondria privileged communication, which might be caused by the increased proximity between both organelles. Yet, this was independent of resting mitochondrial Δψ_m_ and mtCU protein levels. RNA-seq data has shown that mtCU components expression levels were unaltered in Opa1 KO lymphocytes (Corrado et al., 2021). In addition, silencing of Micu1 increases resting matrix [Ca^2+^]_mito_ (Mallilankaraman et al., 2012; Liu et al., 2016; Gottschalk et al., 2019), as we observe in *Opa1* ^*−/−*^ cells. Given that Opa1 can physically interact with Mcu-Micu1 complex (Tomar et al., 2019; Herkenne et al., 2020), it remains possible that the absence of Opa1 might alter Micu1 homo or heterodimerization with Micu2, which have been proposed to control Ca^2+^_mito_ uptake threshold (Patron et al., 2014). Also, it has been suggested that loss of cristae junction can expose a population of mtCU that is not protected by the gatekeeper Micu1 in the cristae lumen (Gottschalk et al., 2019), this explanation could justify the increased resting a maximal Ca^2+^_mito_ observed in the Opa1 KO cells. However, this explanation would not be consistent with a lack of leftward shift in the [Ca^2+^]_mito_ rise during SOCE.

How does OPA1 influence ER-mitochondrial Ca^2+^ transfer? It has been shown that upon agonist stimulation, mitochondrial cristae and matrix modify their volume in HepG2 cells (Booth et al., 2016). Studies in HeLa cells proposed that *OPA1* knockdown decelerates cristae membrane movements. Interestingly, upon IP3R activation, cristae stop their motion at the ER-mitochondria contact region, in an OPA1 independent manner (Gottschalk et al., 2018). Thus, it remains a question whether perturbation in OPA1 is connected to cristae dynamics adaptations associated with Ca^2+^_mito_ uptake.

Regarding the role of OPA1 in Ca^2+^_mito_ uptake in response to bulk Ca^2+^_cyto_ induced by SOCE, our data are opposed to previous studies in HeLa and H295R *OPA1* silenced cells performed in permeabilized cells and depolarized mitochondria conditions (Fülöp et al., 2011). These discrepancies can be attributed to the experimental setting and the temporary vs. long-term OPA1 ablation conditions. As oppose to the cited work, our experimental conditions involve polarized mitochondria and chronic Opa1 ablation. In addition, we found that upon SOCE, WT *OPA1* rescue in *Opa1* ^*−/−*^ cells restored the Ca^2+^_cyto_-dependence of the Ca^2+^_mito_ uptake even beyond that in the WT cells. The exact restoration of the Ca^2+^_mito_ homeostasis in the *Opa1* ^*−/−*^ cells likely requires more than one OPA1 isoforms, as it has been demonstrated for mitochondrial morphology (Del Dotto et al., 2017). Thus, we propose that OPA1 plays distinctive roles in bulk versus ER-to-mitochondria-driven Ca^2+^_mito_ uptake.

Our results showed that all the ADOA patients’ cells that exhibited low levels of OPA1 displayed closer ER-mitochondria contacts, independent of the mutated domain. In a previous study in skin fibroblast from patients carrying a splicing defect at the GTPase domain, cells exhibited an enhanced Ca^2+^_mito_ uptake rate (Fülöp et al., 2015). However, the GTPase mutants explored in this study, both in patient’s cells and acutely expressed in MEFs, displayed no changes in [Ca^2+^]_mito_. Interestingly, the previous study found a high dispersion between patients carrying the same mutation (Fülöp et al., 2015), suggesting that part of the effect could be related to environmental adaptations or the patient’s genetic background.

To isolate the domain-specific mutants’ effect we performed *in vitro* studies in *Opa1* ^*−/−*^ background. We found that the integrity of the whole OPA1 protein is necessary to restore resting [Ca^2+^]_cyto_, given that neither GTPase nor GED mutants altered resting [Ca^2+^]_cyto_. The GTPase domain was not required to restore ER-dependent or ER-independent [Ca^2+^]_mito_ uptake. Previous studies have shown that GTPase is critical for mitochondrial fusion activity (DeVay et al., 2009; Del Dotto et al., 2017), suggesting that the OPA1 Ca^2+^ role can be independent of the fusion activity. Instead, the GED mutants couldn’t rescue resting or maximal [Ca^2+^]_cyto_, whereas, resting but no maximal [Ca^2+^]_mito_ was augmented. Elevated resting [Ca^2+^]_mito_ was also found in the patient’s cells harboring the *OPA1* GED mutant c.2713C>T, suggesting that this effect could be specific for this domain. In addition, the GED mutants did not restore the [Ca^2+^]_cyto_ needed to induce [Ca^2+^]_mito_ rise, suggesting that the OPA1 GED domain is essential for the maintenance of resting Ca^2+^_mito_ and Ca^2+^_mito_ uptake mediated by ER-mitochondria Ca^2+^ transfer. OPA1 GED domain is a predicted coiled-coil domain (Akepati et al., 2008); however, no molecular partner, except for SIRT3, has been linked to physically interact with this domain (Samant et al., 2014). Strikingly, GED mutant’s expression restored bulk [Ca^2+^]_cyto_ needed to induce [Ca^2+^]_mito_ rise, proposing that OPA1 GED domain could be relevant during ER-mitochondria functional tethering through a, so far, unknown molecular partner.

Finally, ADOA is a dominant disease, most of the patients are heterozygous *OPA1* mutant, with only a few reports of patients with homozygous *OPA1* mutations (MIM #605290). The inclusion of *OPA1* GTPase or GED mutants in the presence of WT Opa1 in MEF cells, slightly diminished the Ca^2+^_mito_ uptake without alteration of Mcu levels. A leftward shift in [Ca^2+^]_cyto_ dependence to promote [Ca^2+^]_mito_ rise was a particular characteristic observed in the *Opa1* ^*−/−*^ cells. Moreover, ADOA patient cells exhibiting low levels of OPA1 displayed a closer ER-mitochondria distance, suggesting that OPA1 integrity and levels are necessary for physiological intracellular Ca^2+^ regulation and ER-mitochondria Ca^2+^ transfer. Thus, the cellular models studied here, with patient cells and the acute expression of *OPA1* mutants in a WT background, all point towards a dominant-negative Ca^2+^ homeostasis phenotype, independent of the involved domain.

In conclusion, our study provides new evidence on a central role for OPA1 in Ca^2+^ homeostasis and in determining functional ER-mitochondrial coupling, with the GED region playing a fundamental role in stabilizing the efficiency of inter-organelle Ca^2+^ transfer. The co-existence of WT and ADOA-related mutants could perturb ER-to-mitochondrial communication, providing a mechanism for disease progression in patients affected with ADOA disease.

## Materials and Methods

### Cell Culture

Experiments were performed in fibroblasts derived from ADOA patients and control individuals or WT and Opa1 KO Mouse embryonic fibroblasts (MEFs) (provided by David Chan). Patient-derived cells were provided by Newcastle Research Biobank for Rare and Neuromuscular Diseases, based on a Material Transference Agreement with Pontificia Universidad Católica de Chile, and were maintained following the institutional biosecurity and bioethics protocols. All the cells were cultured in high glucose Dulbecco-Eagle modified medium containing sodium pyruvate (DMEM, Gibco Cat#1280017) and supplemented with 10% of fetal bovine serum (FBS), 2 mM glutamine, and 100 U/ml penicillin, and 100 μg/ml streptomycin in humidified air (5% CO_2_) at 37°C. Given that human myoblasts grow slowly, skeletal muscle-derived fibroblasts were generated from myoblast samples. Briefly, the patient’s derived myoblasts were cultured in Skeletal Muscle Cell Growth Medium (PromoCell #CatC-23160) and plated in collagen I-coated dishes. Cells were trypsinized and pre-plated in an uncoated dish for 30 min to separate fibroblasts from myoblasts, where the former adhere to the uncoated dish while the latter were resuspended and placed aside. The patient’s fibroblasts were used between passages 3–9. All the cells were tested for *mycoplasma* contamination regularly using the following protocol (Young et al., 2010).

### Cell Transfection

Cells were plated on glass 25 mm coverslips and then transfected with specific constructs using Lipofectamine 2,000–3,000 (Invitrogen). MEF cells were transfected pCCEY plasmid encoding for human OPA1 isoform 1. OPA1 GTPase or GED mutants were built in the same backbone. For mitochondrial matrix calcium analysis, we used mitochondrial matrix-targeted Ca^2+^-sensitive proteins mtRCaMP (K_d_ ∼ 1 µM) and CEPIA3mt (K_d_ ∼ 10 µM). The transfection was performed with OPTI-mem (Thermofisher) or Transfectagro (Corning) and lipofectamine 2,000 or 3,000 (Invitrogen, Cat#11668019 or Cat#L300015, respectively) according to the manufacturer´s protocol. For optimal expression, the cells were grown for 48 h after transfection. One μg of DNA per plasmid per 35 mm dish was used for each experiment.

### Ca^2+^ Measurement

MEFs were transfected with the mtRCaMP, the muscarinic receptor type 3 (M3R), and OPA1 mutants. Forty-eight hours after transfection the cells were incubated in a serum-free extracellular medium (ECM: 121 mM NaCl, 5 mM NaHCO3, 10 mM Na-HEPES, 4.6 mM KCl, 1.2 mM KH2PO4, 1.2 mM MgSO4, 2 mM CaCl2, 10 mM glucose, pH7.4) containing 2%BSA and loaded with Fura2-AM (2 µM) in presence of 0.0003% Pluronic F-127 and 100 μM sulfinpyrazone for 15 min at room temperature. Cells were washed once with ECM containing 2% BSA, and recorded in ECM containing 0.25% BSA, and transferred to the thermostated stage (37°C) of the microscope. Cells were stimulated by Carbachol (CCh) 200 nM to activate the M3R, and subsequently, the IP3R, to induce Ca^2+^ release from the ER. For SOCE, after Fura2-AM incubation, cells were washed once with a Ca^2+^ free buffer. Imaging was performed in a Ca^2+^-free ECM containing 0.25% BSA. Cells were stimulated by CCh 200 nM to activate the IP3R and induce Ca^2+^ release from the ER and recorded for 150s, then the cells were stimulated with Thapsigargin 2 µg to deplete the ER of Ca^2+^ and recorded for 300s, and finally, 1 mM of Ca^2+^ was added to induce SOCE. Images were acquired using an ImagEM EM-CCD camera (Hamamatsu) fitted to an Olympus IX81 microscope with LED source (Lambda TLED+, Sutter Instruments). The configuration for each fluorescent probe or protein was as follow: Fura2-AM was recorded using 340–380 nm excitation; mtRCaMP was recorded with a 577 nm excitation filter; CEPIA3mt was recorded using 485 nm excitation filter (Chroma, customized 59022 UV filters), using dual-band dichroic and emission filters (Chroma, 59022m dual-band filters). Image collection frequency, 1 Hz. Calibration of the Fura2-AM signal was carried out at the end of each measurement, adding 1 mM CaCl_2_, followed by 10 mM EGTA/Tris, pH 8.5. The Fura2-AM ratios were calibrated in terms of nM [Ca^2+^]_cyto_ using the following formula:

$${\left[{\text{Ca}}^{2+}\right]}_{\text{cyto}}\;\left(\text{nM}\right)={\text{K}}_{d\;\text{Fura}2\text{AM}}\;\times \;\left[\frac{\text{R}-{\text{R}}_{\text{min}}}{{\text{R}}_{\text{max}}-\text{R}}\right]\times \left[\frac{{\text{F}}_{\text{max}}^{380}}{{\text{F}}_{\text{min}}^{380}}\right]$$

Where Fura2-AM K_d_ was 224 nM, R is ratio 340/380 nm, R_min_ is the ratio 340/380 after EGTA addition, R_max_ is the ratio 340/380 nm after 1 mM CaCl_2_ addition, F_max_ is the maximum 380 nm fluorescence upon EGTA addition. F_min_ is the minimum 380 nm fluorescence upon 1 mM CaCl_2_ addition. Maximal [Ca^2+^]_mito_ uptake was calculated as F_max_/F_0_ where F_max_ is the maximum fluorescence intensity and F_0_ is the mean resting fluorescence intensity before IP3R stimulation or SOCE protocol induction.

For [Ca^2+^]_mito_ vs. [Ca^2+^]_cyto_ analysis we plotted the mean trace curve from basal to maximal [Ca^2+^]_cyto_ in X-axis, with regards to the same time points corresponding to [Ca^2+^]_mito_ relative fluorescence from basal to maximal [Ca^2+^]_cyto_, in the Y-axis. For maximal IP3R-induced [Ca^2+^]_cyto_ vs maximal [Ca^2+^]_mito_ uptake we used the maximal [Ca^2+^]_cyto_ ± SEM in the X-axis, and the maximal [Ca^2+^]_mito_ uptake ±SEM, in the Y-axis. To quantify the changes in the [Ca^2+^]_mito_ vs. [Ca^2+^]_cyto_ we consider considered the [Ca^2+^]_cyto_ at the moment of a 2-fold increase in [Ca^2+^]_mito_ upon CCh stimulation. For SOCE, we considered the [Ca^2+^]_cyto_ at the moment of a 1.5-fold increase in [Ca^2+^]_mito_ for CCh stimulation.

For ADOA-derived fibroblasts experiments, the cells plated on glass coverslips were transfected with mtRCaMP and loaded with Fura2-AM (2 µM). Cells were evaluated in 0.25% BSA-ECM containing and transferred to the thermostated stage (37°C) of the microscope. Live-cell image series was performed every 1.5s, in a Nikon Eclipse Ti inverted microscope with a Lambda DG4 wavelength-switch xenon light source (Sutter Instruments) and attached by iXon3 897 Andor EMCCD camera controlled by NIS software (Nikon). Cells were stimulated by Histamine to activate the IP3R and induce ER Ca^2+^ release. Fura2-AM was evaluated by a dedicated filter set cube: ex.340,380—em.535/40 nm, whereas, mtRCaMP was tested using ex.545—em.620/60 nm Ca^2+^_cyto_ transient data were expressed as 340/380 ratio. To quantify the level of change in the [Ca^2+^]_mito_ vs. [Ca^2+^]_cyto_ we considered the [Ca^2+^]_cyto_ at the moment of a 1.4-fold increase in [Ca^2+^]_mito_ upon Histamine stimulation.

### Mitochondrial Membrane Potential

Cells plated on glass coverslips were washed with ECM and loaded 10 min at room temperature with Tetramethyl rhodamine methyl ester or Tetramethyl rhodamine ethyl ester probe (TMRM 20 nM for MEF cells or TMRE 10 nM for human fibroblasts) in non-quenching mode. Mitochondrial membrane potential in MEF cells was measured at the custom-built LED Olympus epifluorescence imaging system. TMRM was recorded using a 577 nm excitation filter, using dual-band dichroic and emission filters (Chroma, 59022m dual-band filters). Cells were recorded every 1s. ADOA-derived cells experiments were performed at the Nikon Eclipse Ti microscope, using the following configuration: ex. 540 nm—em. 620/60 nm, 1 image every 1.5s. FCCP 5 µM/Oligomycin 5 μg/ml (MEF cells) or FCCP 10 µM (ADOA-derived fibroblasts) was used to induce loss of mitochondrial membrane potential. Resting membrane potential was calculated as ΔF_basal_—F_FCCP_ using absolute fluorescence values.

### Transmission Electron Microscopy

To evaluate mitochondrial ultrastructure, pellets of 8 × 10^5^ cells were fixed with glutaraldehyde 2.5%. Staining was performed as previously described (Csordás et al., 2010). Images of ultra-thin sections were acquired in a transmission electron microscope Philips Tecnai 12 at 80 kV, or at a TALOS F200C G2 system (Thermo Scientific), equipped with a Ceta 16M CMOS camera, at 200 kV, at the Advanced Microscopy Facility UMA-UC, Pontificia Universidad Católica de Chile. ER-mitochondria distance analysis was performed with Fiji ImageJ. For ER-mitochondria distance measurement we considered the values ≤30 nm.

### Western Blot Analysis

Cells were cultured to 70–80% of confluence, harvested, and frozen. Upon thawing, a membrane-rich lysate was generated by RIPA buffer supplemented with protease and phosphatase inhibitors; 30 µg of total protein extracts were loaded into 8% or 4–12% gradient SDS-PAGE gel and transferred to PVDF membranes. Membranes were blocked with 5% milk in 0.1% TBS-Tween for 1 h at RT, followed by overnight incubation with primary antibody prepared in 5% milk or 3% BSA in 0.1% TBS-Tween. Secondary antibodies were visualized with enhanced chemiluminescent substrates (ECL, SuperSignal West Dura or SuperSignal West Femto, Thermo Scientific). For MICU1, MICU2, and EMRE, were used IR LI-COR Bioscience secondary antibodies. Densitometry was performed using ImageStudio software (LI-COR Bioscience). Antibodies used in this study: OPA1(1:1,000, BD Pharmigen #Cat 612607), MICU1 (1:500, Sigma #Cat HPA037479), MICU2 and EMRE (1:1,000, Bethyl) MCU (1:1,000, Cell signaling #Cat14997, 1:500 Sigma-Aldrich #Cat HPA016480), mtHSP70 (1:1,000 Invitrogen, #Cat MA3-028), TIM23 (1:1,000, BD Pharmigen #Cat 611-222), TOM20 (1:1,000, Proteintech #Cat 11802-1-AP), TUBULIN (1:1,000 Cell Signaling #Cat 2144S), GAPDH (1:5,000, Proteintech #Cat 60004-1-lg).

### OPA1 Domain-specific Mutations Plasmid Construction

The pCCEY plasmid containing human OPA1 isoform 1 WT was kindly donated by Guy Lenaers. Mutagenic OPA1 variants were designed using the Q5 Site-Directed Mutagenesis Kit (New England Biolabs) and following the manufacturer´s protocol. The actual generation of specific mutations was confirmed by sequencing using the ABI PRISM 3500 xl Applied Biosystems (FONDEQUIP EQM150077) at Pontificia Universidad Católica de Chile or sequenced at Macrogene inc., Seoul, South Korea. pCCEY plasmid encoding GTPase mutant *OPA1* c.899G>A (G300E) and GED mutant *OPA1* c.2708delTTAG was kindly donated by Guy Lenaers and used in previous studies (Olichon et al., 2007b; Eisner et al., 2014). For primers’ details, see Table 1.

**TABLE 1:** Primers used to build the mutants generated in this work.

Mutant	Primers
*OPA1* c.870+5G>A	5′ GTT​GTT​GTG​GTT​GGA​GAT​C 3′
	3′ CTT​AAG​CTT​TCT​ATG​ATG​AAT​G 5′
*OPA1* c.889C>T	5′ GGT​TGG​AGA​TtA​GAG​TGC​TGG 3′
	3′ ACAACAACCCGTGGCAGA 5′
*OPA1* c.1334G>A	5′ GAT​GCT​GAA​CaC​AGT​ATT​GTT​AC 3′
	3′ CAC​AGA​TCC​ATC​TTG​AAT​AC 5′
*OPA1* c.2713C>T	5′ TGA​AGT​TAG​GtG​ATT​AGA​GAA​AAA​TG 3′
	3′ GTA​TTT​GTA​AGT​TGT​TGC​C 5′
*OPA1* c.2818+5G>A	5′ AGA​AAG​TTA​GAG​AAA​TTC​AAG​AAA​AAC 3′
	3′ CTT​CAG​TAT​TTG​TAA​GTT​GTT​G 5′

### Stable Cell Line Generation

*Opa1* ^*−/−*^ MEFs cells were transfected as described above with a lentiviral plasmid containing the human OPA1 isoform 1 and a puromycin resistance cassette (pLenti-OPA1). Transfected cells were selected with Puromycin 2 μg/ml to obtain a polyclonal stable cell line. The cells were grown in growth media described above in presence of Puromycin 2 μg/ml. The pLenti-OPA1 was obtained from VectorBuilder.

### Image and Statistical Analysis

Image acquisition and analysis were performed using Fiji (ImageJ) software. The statistical analysis was carried out using the GraphPad Prism 8 Software. For pairwise comparisons, unpaired *t*-tests were used for all normally distributed data, whereas Mann-Whitney tests were used for nonparametric data. For multiple comparisons, One-way ANOVA followed by Dunnett’s multiple comparison test was used to determine the significance of normally distributed data. For nonparametric multiple comparisons, a Kruskal-Wallis test followed by Dunn’s multiple comparison test was used to determine significance (*p* < 0.05). In all cases, data not indicated as significant should be considered not statistically different.

## Data Availability

The original contributions presented in the study are included in the article/Supplementary Material, further inquiries can be directed to the corresponding authors.

## Supplementary Material

Supplementary Figure 1
**(A)** *Opa1* ^*−/−*^ *, Opa1* ^*−/−*^ *+OPA1* WT or WT MEF cells expressing mtRCaMP, and the muscarinic receptor 3 (M3R), were incubated with Fura2-AM as described above, and stimulated with 200 µM of Carbachol (CCh) to induce the release of Ca^2+^ from the ER to the cytosol via activation of IP3R. The graph shows resting [Ca^2+^]_cyto_. **(B)** Graph show mean traces of [Ca^2+^]_mito_ expressed in arbitrary fluorescence units for mtRCaMP. **(C)** Resting [Ca^2+^]_mito_ expressed in arbitrary fluorescence units. **(D)** *OPA1* ^*−/−*^ MEF cells exogenously expressing CEPIA3mt, M3R, and incubated with TMRM 20nM as described above, were stimulated with 200 µM of Carbachol to induce the release of Ca^2+^ from the ER to the cytosol via activation of IP3R. Graph show mean traces of [Ca^2+^]_mito_ expressed in arbitrary fluorescence units (WT, n=3/99; preparations/cells; *Opa1* ^*−/−*^, *n* = 3/103; *Opa1* ^*−/−*^ *+OPA1* WT, *n* = 3/99). **(E)** Resting [Ca^2+^]_mito_ expressed in arbitrary fluorescence units. **(F)** Maximal [Ca^2+^]_mito_ uptake vs Maximal [Ca^2+^]_cyto_ amplitude upon agonist stimulation of WT single cells. **(G)** Maximal [Ca^2+^]_mito_ uptake vs Maximal [Ca^2+^]_cyto_ amplitude upon agonist stimulation. **(H)** *Opa1* ^*−/−*^ *, Opa1* ^*−/−*^ *+OPA1* WT or WT MEF cells expressing OMMRCaMP were washed and kept in a Ca^2+^-free medium. The cells were treated 2µM of Thapsigargin (Tg) (250s) to deplete the ER Ca^2+^ content. The graph shows mean traces of [Ca^2+^]_cyto_ (WT, n=2/40; preparations/cells; *Opa1* ^*−/−*^, n=2/72; *Opa1* ^*−/−*^ +*OPA1*WT, n=2/49). **(I)** [Ca^2+^]_OMM_ mean traces upon Tg addition. **(J)** AUC from [Ca^2+^]_cyto_ traces **(K)** AUC from [Ca^2+^]_OMM_ traces **(L)** Store Operated Calcium Entry (SOCE) induction protocol. Prior to stimulation with CCh, cells were washed and kept in a Ca^2+^-free medium. To stimulate IP3R, cells were treated with CCh 200 µM (60s), followed with 2 µM of Tg (200s) to completely deplete the Ca^2+^ from the ER. Next, 1mM of CaCl_2_ was added to induce the Store Operated Calcium Entry (SOCE, 500s). The graph shows mean traces of [Ca^2+^]_cyto_. **(M)** Maximal [Ca^2+^]_cyto_ amplitude upon CCh stimulation in Ca^2+^-free buffer. **(N)** Maximal [Ca^2+^]_cyto_ amplitude upon Tg stimulation in Ca^2+^-free buffer. **(O)** Maximal [Ca^2+^]_mito_ uptake vs Maximal [Ca^2+^]_cyto_ amplitude upon SOCE induction. Data are mean ± SEM. Error bars represent SEM. **p* < 0.05, ***p* < 0.01, ****p* < 0.001, *****p* < 0.0001 vs. WT condition. ^####^ *p* < 0.0001 vs. *Opa1* ^*−/−*^ + *OPA1* WT condition.

Supplementary Figure 2
**(A)** Western blot analysis of mitochondrial mass markers for different mitochondrial compartments (Matrix, IMM, OMM). **(B)** Western blot densitometry of mitochondrial mass markers for different compartments (Matrix, IMM, OMM). **(C)** Western blot analysis of mtCU components in WT, *Opa1* ^*−/−*^or acute rescued *Opa1* ^*−/−*^ MEF Cells. **(D)** Western blot densitometry of main mtCU components. **(E)** Western blot validation of *Opa1* ^*−/−*^ +*OPA1* WT stable MEFs cells. Data are mean ± SEM. Error bars represent SEM. **p* < 0.05 vs. WT.

Supplementary Figure 3
**(A)** Western blot quantification of OPA1 levels. **(B)** Skin or skeletal muscle ADOA-derived fibroblast expressing mtRCaMP were loaded with Fura2-AM (2 μM) for 20 min at room temperature. To induce the release of Ca^2+^ through IP3R activation, Histamine 100 μM was used. The bar chart shows resting [Ca^2+^]_cyto_. **(C)** The bar chart shows resting [Ca^2+^]_mito_. **(D)** Maximal [Ca^2+^]_mito_ uptake vs Maximal IP3R-induced [Ca^2+^]_cyto_ amplitude upon agonist stimulation. **(E)** TEM representative images of ER-mitochondria contacts from ADOA-derived patient cells. ER is pseudo-colored in blue. Data are mean ± SEM. Error bars represent SEM. ***p* < 0.01, ****p* < 0.001 vs. Control condition.

Supplementary Figure 4
**(A)** Western blot quantification of OPA1 levels. **(B)** Skin or skeletal muscle ADOA-derived fibroblast expressing mtRCaMP were loaded with Fura2-AM (2 μM) for 20 min at room temperature. To induce the release of Ca^2+^ through IP3R activation, Histamine 100 μM was used. The bar chart shows resting [Ca^2+^]_cyto_. **(C)** The bar chart shows resting [Ca^2+^]_mito_. **(D)** Maximal [Ca^2+^]_mito_ uptake vs Maximal IP3R-induced [Ca^2+^]_cyto_ amplitude upon agonist stimulation. **(E)** TEM representative images of ER-mitochondria contacts from ADOA-derived patient cells. ER is pseudo-colored in blue. Data are mean ± SEM. Error bars represent SEM. ***p* < 0.01, ****p* < 0.001 vs. Control condition. **(F)** Maximal [Ca^2+^]_mito_ uptake vs Maximal [Ca^2+^]_cyto_ amplitude induced upon agonist stimulation. **(G)** Maximal [Ca^2+^]_mito_ uptake vs Maximal [Ca^2+^]_cyto_ upon SOCE induction. **(H)** WT MEF cells expressing OPA1 mutants, mtRCaMP, and the muscarinic receptor 3 (M3R), were incubated with Fura2-AM and stimulated as described above. Graph shows mean traces of [Ca^2+^]_cyto_ expressed in nM. The bar chart shows resting [Ca^2+^]_cyto_. **(I)** Graphs show mean traces of [Ca^2+^]_mito_ expressed in arbitrary fluorescence units, evoked by GTPase mutants. **(J)** Graphs show mean traces of [Ca^2+^]_mito_ expressed in arbitrary fluorescence units, evoked by GED mutants. **(K)** The bar chart shows resting [Ca^2+^]_mito_. **(L)** Maximal [Ca^2+^]_mito_ vs Maximal [Ca^2+^]_cyto_ amplitude induced upon agonist stimulation. Data are mean SEM. Error bars represent SEM. ∗*p* < 0.05, ∗∗*p* < 0.01 vs Opa1−/− or WT condition. #*p* < 0.05, ##*p* < 0.01 vs *Opa1^−/−^* + OPA1 WT condition. The blue color is indicative of GTPase mutants and the red color for GED mutants.
