# Supplementary material for: OPA1 Modulates Mitochondrial Ca2+ Uptake Through ER-Mitochondria Coupling
Source: Front Cell Dev Biol. 2022 Jan 3;9:774108. doi: 10.3389/fcell.2021.774108 (PMC8762365; doi:10.3389/fcell.2021.774108)
Supplement: Supplementary file 1 [file Table1.docx]

Supplementary Table 1: Data summary of main findings in the different model systems

|  | **ER-Mito Association** | **CCh [Ca^2+^]_cyto_ vs [Ca^2+^]_mito_** | **SOCE [Ca^2+^]_cyto_ vs [Ca^2+^]_mito_** |
| --- | --- | --- | --- |
| WT | - | - | - |
| *Opa1^-/-^* | Increased | Leftward shift | Rightward shift |
| *Opa1^-/-^* + OPA1 | Rescued | Partial rescue | Rescued |
| **ADOA Patients’ cell** | | | |
| Control | - | - | ND |
| c.870+5g>a | Increased | Partial leftward Shift | ND |
| c.889c>t | Increased | Partial leftward Shift | ND |
| c.2713c>t | Unchanged | Partial leftward Shift | ND |
| c.2818+5g>a | Increased | ND | ND |
| **OPA1 mutants’ rescue (*Opa1^-/-^* background)** | | | |
| *Opa1^-/-^* | Increased | Leftward shift | Rightward shift |
| +OPA1 WT | Rescued | Partial rescue | Rescued |
| c.870+5g>a | ND | Partial rescue | Rescued |
| c.889c>t | ND | Partial rescue | Rescued |
| c.1334g>a | ND | Partial rescue | Rescued |
| c.2708delTTAG | ND | Not rescued | Rescued |
| c.2713c>t | ND | Not rescued | Rescued |
| c.2818+5g>a | ND | Not rescued | Rescued |
| **OPA1 mutants’ overexpression (WT background)** | | | |
| WT | - | - | - |
| +OPA1 WT | ND | Unchanged | ND |
| c.870+5g>a | ND | Leftward shift | ND |
| c.889c>t | ND | Unchanged | ND |
| c.1334g>a | ND | Leftward shift | ND |
| c.2708delTTAG | ND | Leftward shift | ND |
| c.2713c>t | ND | Unchanged | ND |
| c.2818+5g>a | ND | Leftward shift | ND |

ND: not determined
