# Supplementary figures and images for: OPA1 Modulates Mitochondrial Ca2+ Uptake Through ER-Mitochondria Coupling
Source: Front Cell Dev Biol. 2022 Jan 3;9:774108. doi: 10.3389/fcell.2021.774108 (PMC8762365; doi:10.3389/fcell.2021.774108)

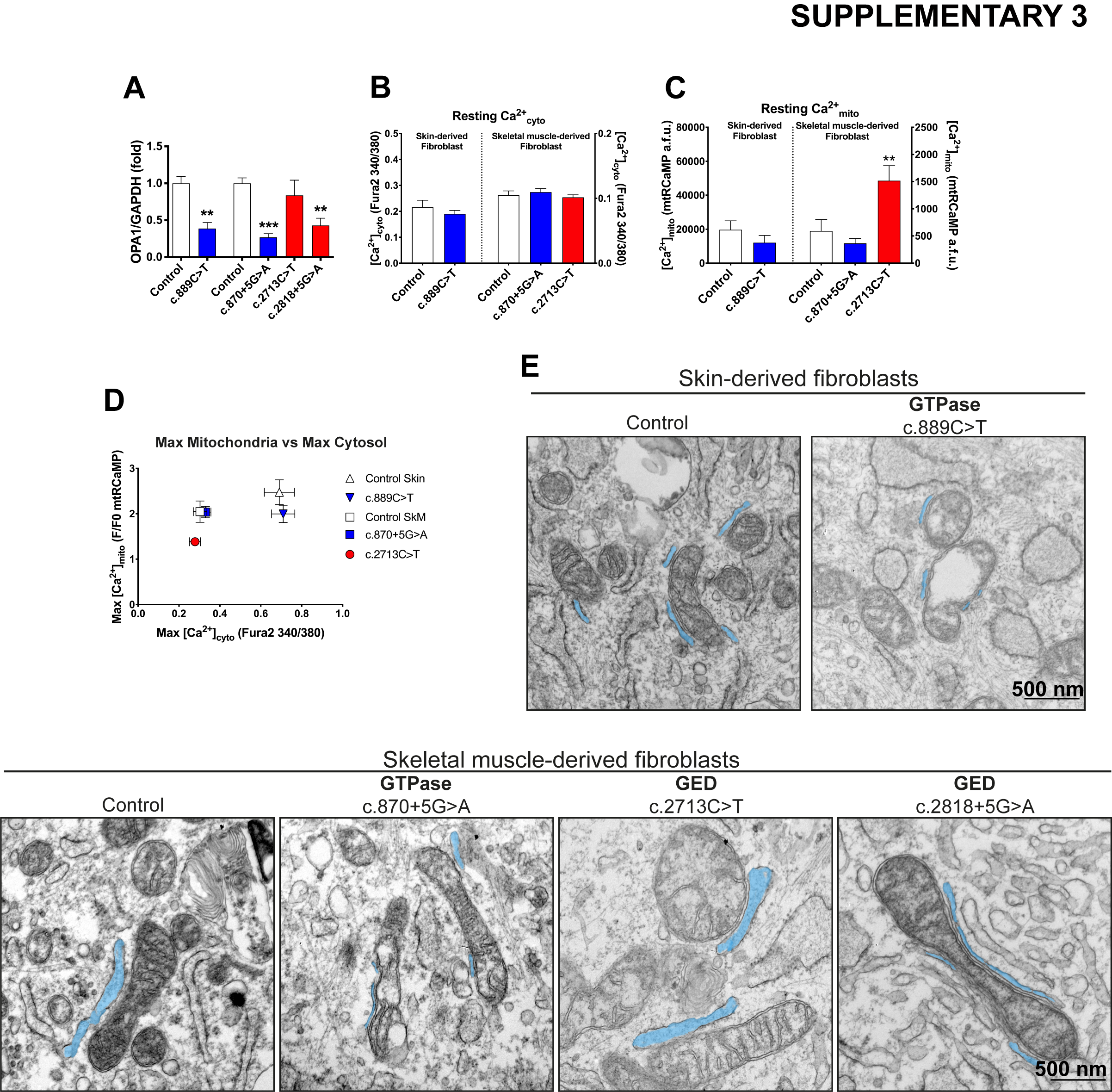

Supplement: Supplementary file 2 [file Image3.TIF]

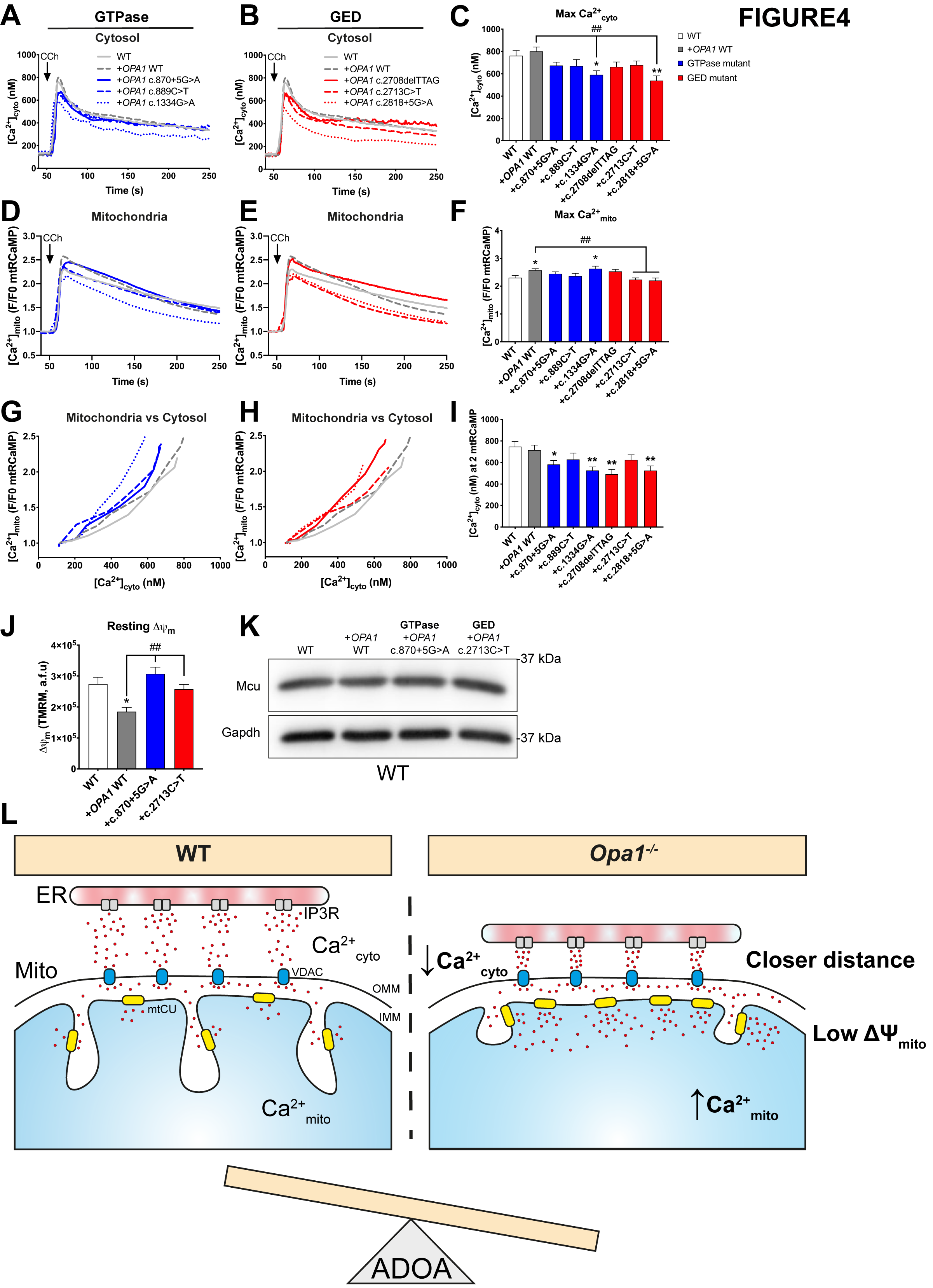

Supplement: Supplementary file 3 [file Figure4.TIF]

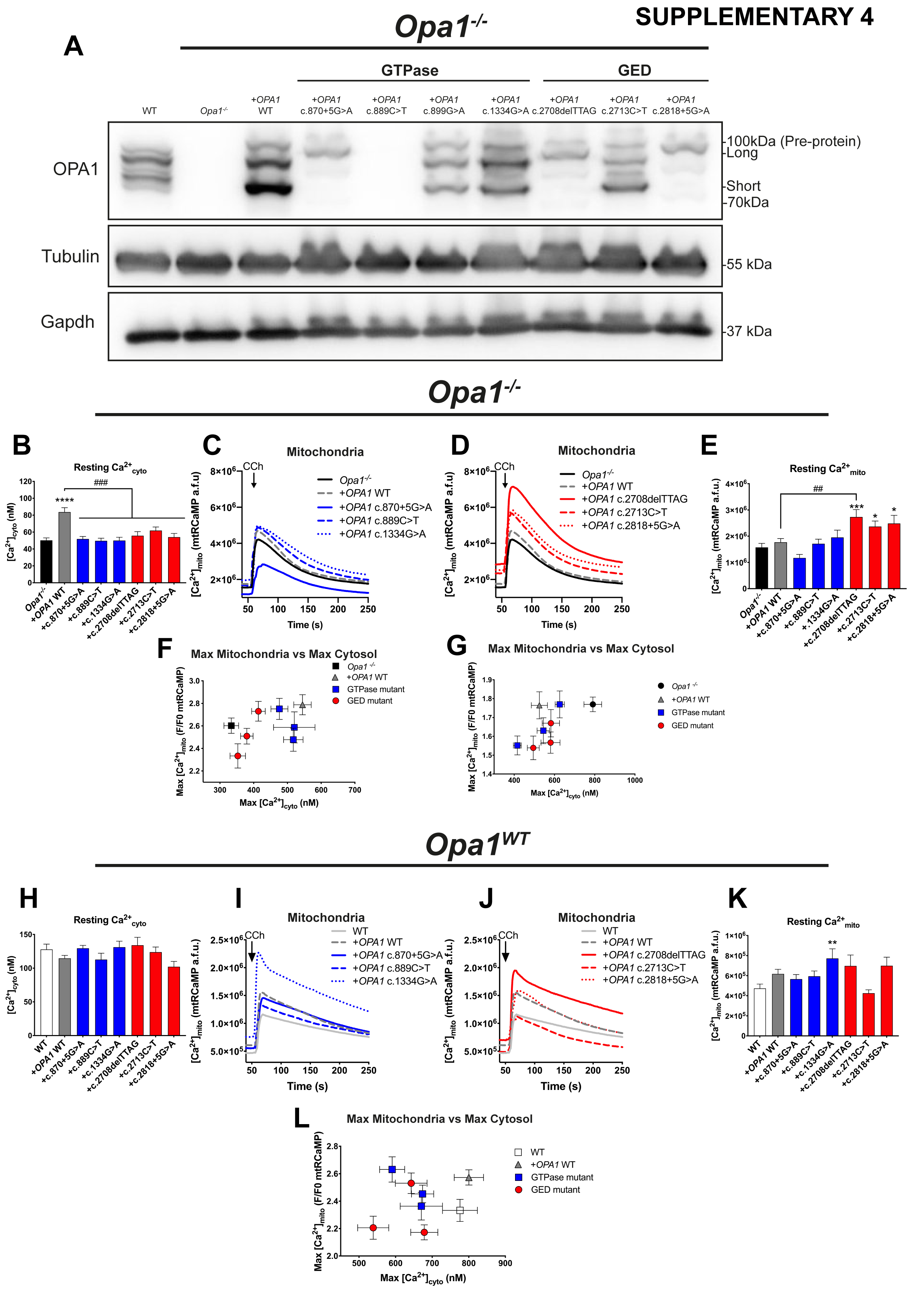

Supplement: Supplementary file 4 [file Image4.TIF]

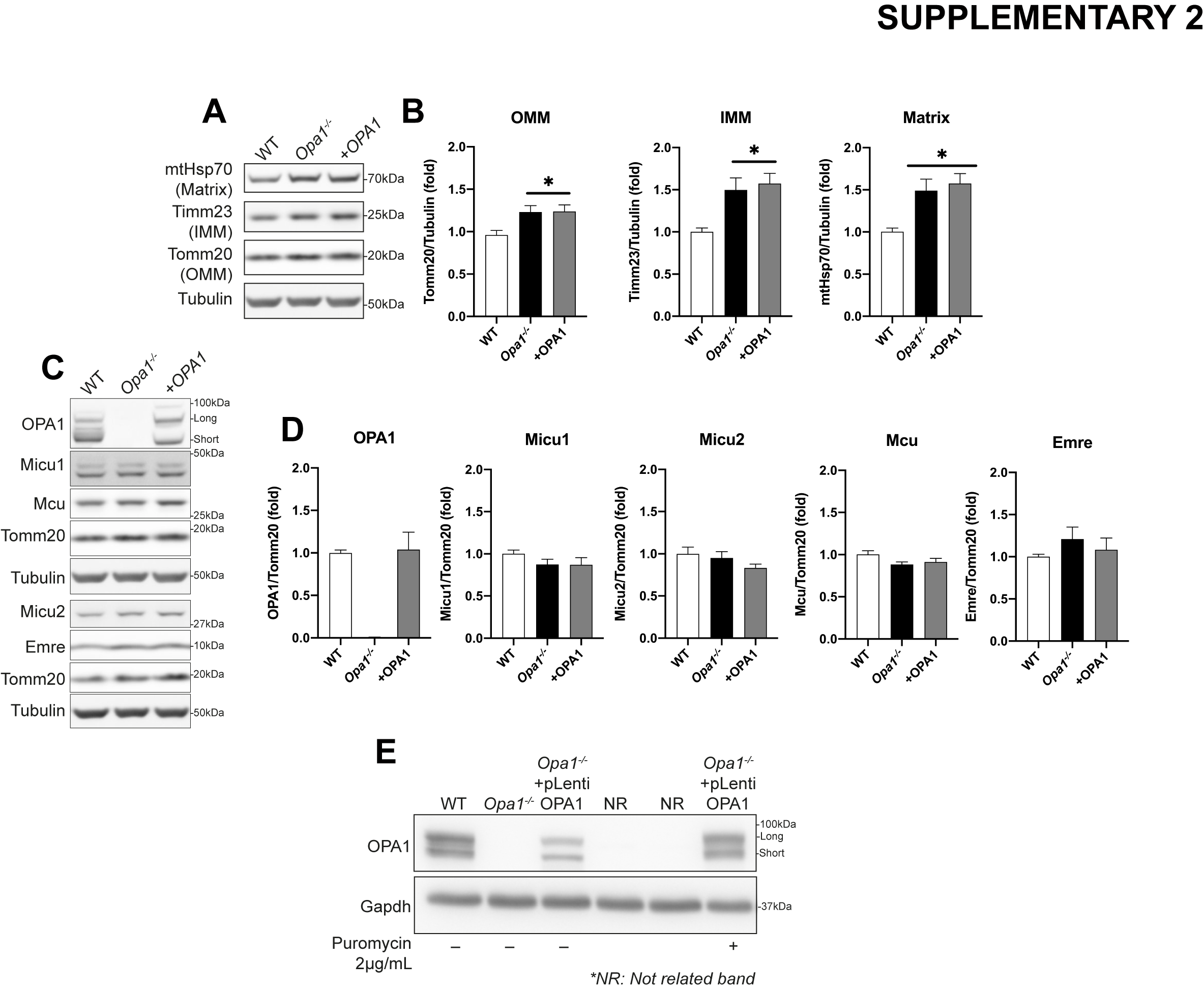

Supplement: Supplementary file 5 [file Image2.TIF]

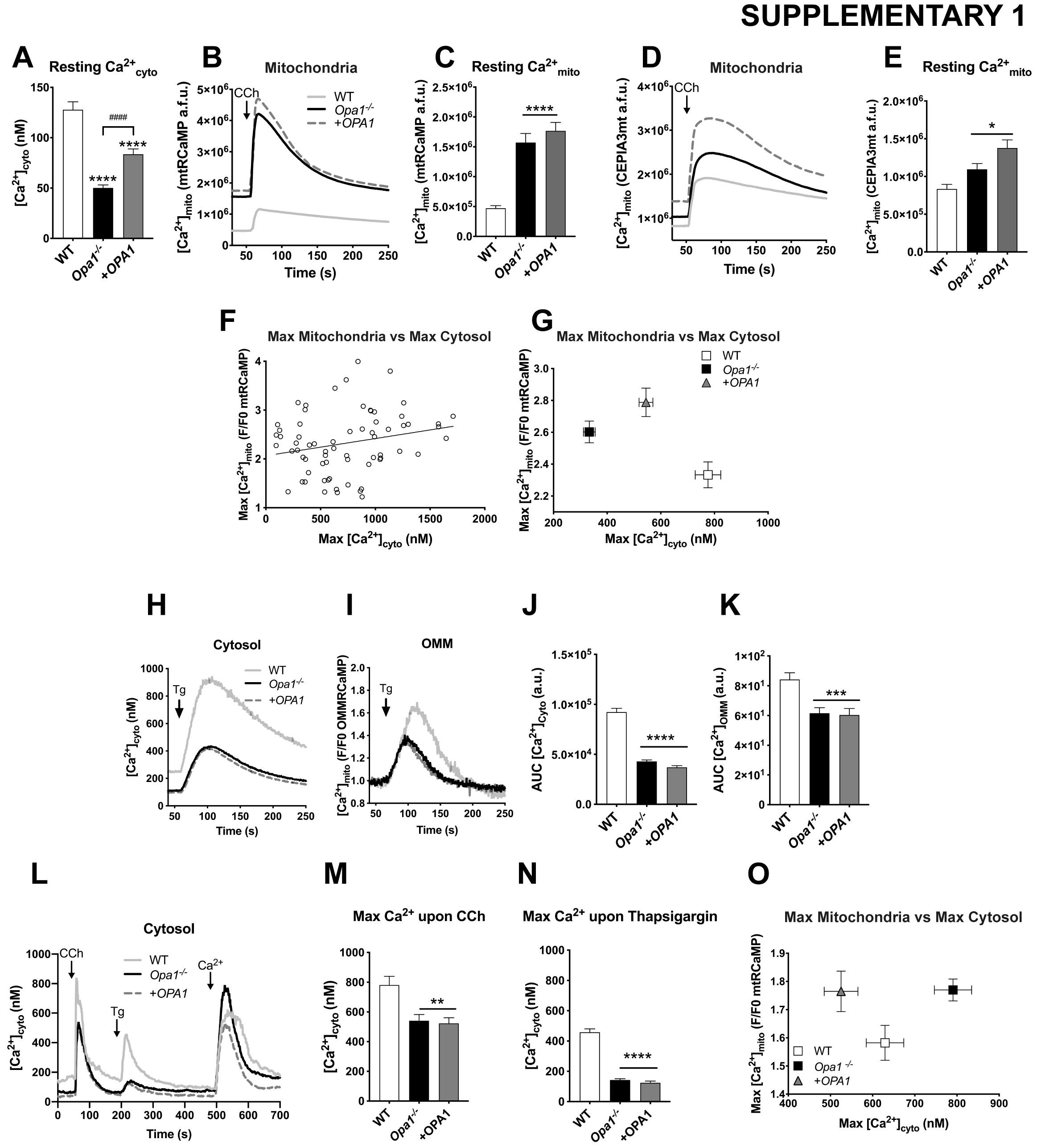

Supplement: Supplementary file 6 [file Image1.TIF]
