# Supplementary material for: OPA1 Modulates Mitochondrial Ca2+ Uptake Through ER-Mitochondria Coupling
Source: Front Cell Dev Biol. 2022 Jan 3;9:774108. doi: 10.3389/fcell.2021.774108 (PMC8762365; doi:10.3389/fcell.2021.774108)
Supplement: Supplementary file 7 [file DataSheet1.PDF]

Supplementary Table 1: Data summary of main findings in the different model systems

	**ER-mito association**	**CCh $[Ca^{2+}]c$ vs mt**	**SOCE $[Ca^{2+}]c$ vs mt**
WT	-	-	-
Opa1$^{-/-}$	Increased	Leftward shift	Rightward shift
Opa1$^{-/-}$ + OPA1	Rescued	Partial rescue	Rescued
Patient cells			
Control	-	-	ND
c.870+5g>a	Increased	Partial leftward Shift	ND
c.889c>t	Increased	Partial leftward Shift	ND
c.2713c>t	Unchanged	Partial leftward Shift	ND
c.2818+5g>a	Increased	ND	ND
Mutants rescue (OPA1 KO MEFs background)			
Opa1$^{-/-}$	Increased	Leftward shift	Rightward shift
+OPA1 WT	ND	Partial rescue	Rescued
c.870+5g>a	ND	Partial rescue	Rescued
c.889c>t	ND	Partial rescue	Rescued
c.1334g>a	ND	Partial rescue	Rescued
c.2708delTTAG	ND	Not rescued	Rescued
c.2713c>t	ND	Not rescued	Rescued
c.2818+5g>a	ND	Not rescued	Not rescued
Mutants overexpression (WT MEFs background)			
WT	-	-	-
+OPA1 WT	ND	Unchanged	ND
c.870+5g>a	ND	Leftward shift	ND
c.889c>t	ND	Unchanged	ND
c.1334g>a	ND	Leftward shift	ND
c.2708delTTAG	ND	Leftward shift	ND
c.2713c>t	ND	Unchanged	ND
c.2818+5g>a	ND	Leftward shift	ND

ND: not determined
